# A molecular design towards sulfonyl aza-BODIPY based NIR fluorescent and colorimetric probe for selective cysteine detection†

**DOI:** 10.1039/d0ra10567h

**Published:** 2021-03-10

**Authors:** Thanh Chung Pham, Yeonghwan Choi, Chaeeon Bae, Cong So Tran, Dongwon Kim, Ok-Sang Jung, Yong-Cheol Kang, SungYong Seo, Hyun Sung Kim, Hwayoung Yun, Xin Zhou, Songyi Lee

**Affiliations:** Department of Chemistry, Pukyong National University Busan 48513 Korea slee@pknu.ac.kr; Industry 4.0 Convergence Bionics Engineering, Pukyong National University Busan 48513 Korea; College of Pharmacy, Pusan National University Busan 46241 Korea hyun@pusan.ac.kr; Department of Chemistry, Pusan National University Busan 46241 Korea; Department of Chemistry, Qingdao University Qingdao 266071 P. R. China zhouxin@qdu.edu.cn

## Abstract

Cysteine (Cys), homocysteine (Hcy) and glutathione (GSH) are essential biothiols for cellular growth, metabolism, and maintenance of a biological system. Thus, the detection of biothiols is highly important for early diagnosis and evaluation of disease progression. In this article, a series of sulfonyl aza-BODIPYs was synthesized, characterized, and examined by ^1^H-NMR, ^13^C-NMR, crystallization, photophysical properties and DFT calculation. Among these structures, a fluorescent probe, BDP-1, exhibited selective detection of Cys among various biothiols *via* nucleophilic aromatic substitution and typical size of Cys molecules. BDP-1 showed color change and near-infrared (NIR) fluorescence enhancement after reaction with Cys to generate BDP-OH, confirmed by HRMS. The red shift of absorption wavelength showed a similar tendency resulting in time-dependent density functional theory (TD-DFT). Furthermore, the calculated detection limit of BDP-1 toward Cys was 5.23 μM. This probe facilitates the colorimetric and fluorescent detection of Cys over other biothiols.

## Abstract 2

A new fluorescent and colorimetric probe based-on sulfonyl aza-BODIPY (BDP-1–3) are designed and synthesized for selective cysteine detection.

## Graphical abstract

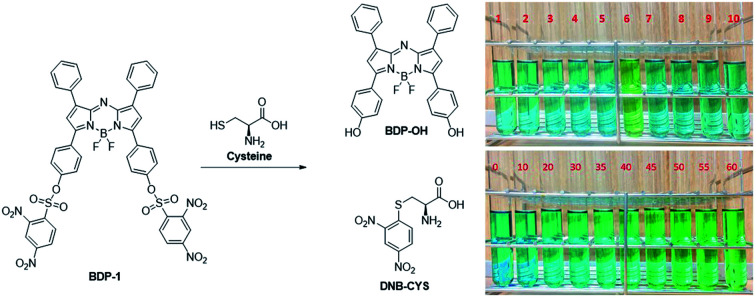

## Introduction

Intracellular thiols, such as cysteine (Cys), homocysteine (Hcy) and glutathione (GSH) play an important role in a wide range of physiological and pathological processes in living organisms.^1,2^ Among various biothiols, the range of free intracellular Cys concentrations varies between 30 and 200 μM.^3,4^ Abnormal levels of Cys are related to several diseases such as hair depigmentation, lethargy, liver damage, muscle and fat loss, and skin lesions.^3,4^ Therefore, it is critical to report changes in Cys concentrations *via* real-time monitoring.

Due to high selectivity, sensitivity, simplicity and fast response, fluorescent probes represent powerful tools to monitor biologically relevant species *in vitro* or *in vivo*. Accordingly, fluorescent probes for Cys, Hcy and GSH have been utilized in molecular recognition or thiol-specific reaction strategies.^5–9^ However, because of the structural similarity of Cys and Hcy, the selective discrimination between the two species is a challenging task. On the other hand, many sulfonyl groups-based probes have been reported with a lack of study compared their substitution ability with biothiols.^10,11^

Aza-boron-dipyrromethene (aza-BODIPY) and its derivatives are a new class of near-infrared (NIR) organic photosensitizers with excellent stability, significant red-shifted absorption and high fluorescence quantum efficiency.^12,13^ The substitution of methine-bridged carbon atom with nitrogen leads to the formation of so-called aza-BODIPY with a bathochromic shift in absorption of about 90 nm with respect to the analogous derivative, which has NIR excitation.^14^ Upon reaction with target molecules, the electron-deficient groups are converted to electron-rich groups, resulting in a different level of absorbance/excitation energy compared with aza-BODIPY. Therefore, aza-BODIPY serves as an ideal base to induce fluorescence emission in NIR. In addition, they have been widely investigated in photovoltaics, imaging, and photodynamic therapy (PDT).^15–19^

In the current study, we synthesized and studied photophysical properties of three sulfonyl aza-BODIPY conjugates (BDP-1–BDP-3). Further, lowest energy structure of aza-BODIPY derivatives were obtained that based on single crystal structure and density functional theory (DFT) calculations. Among them, a near infrared (>700 nm) probe BDP-1 bearing 2,4-dinitrobenzenesulfonyl (DNBS) moieties with stronger nucleophilic aromatic substitution ability compared to toluene- or (dimethylamino)naphthalene-1-sulfonyl groups, which facilitate simultaneous colorimetric and fluorometric detection of Cys over BDP-2 and BDP-3. Compared with the reported NIR fluorescent probes for the recognition of Cys, the present probe exhibits high stability, excellent sensitivity, and significant selectivity.

## Results and discussion

**Fig. 1:**
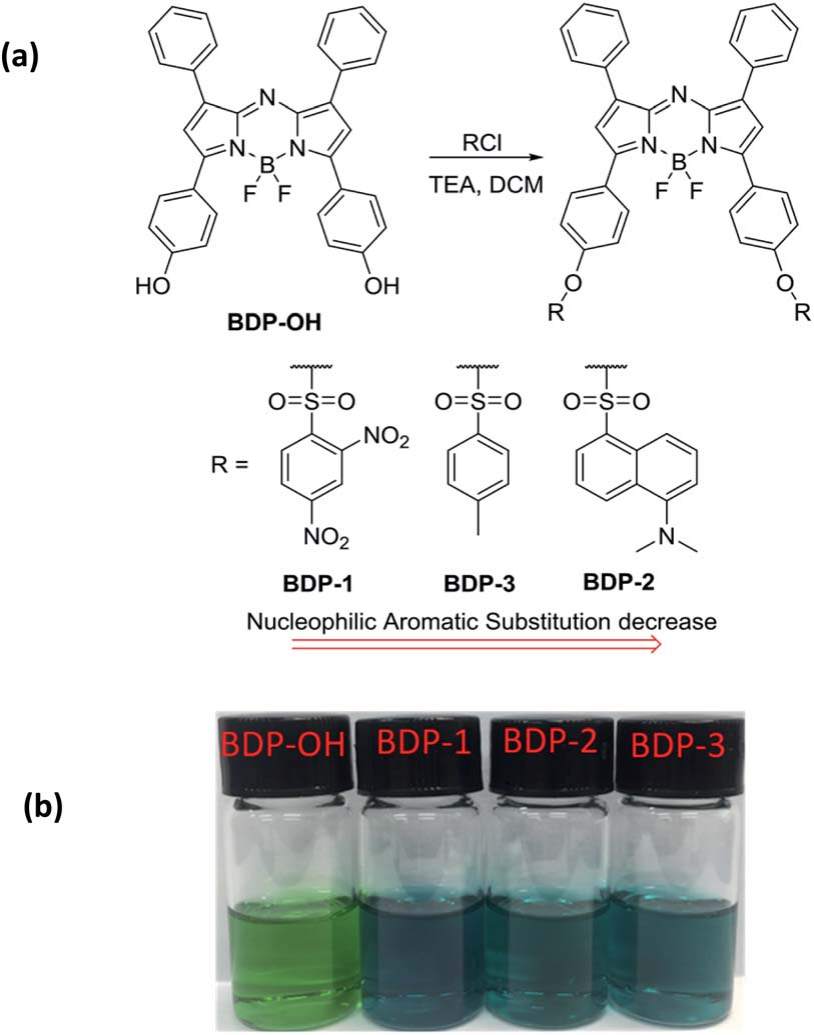
(a) Synthesis process of BDP-1, BDP-2, BDP-3 from BDP-OH. (b) Color of BDP-OH, BDP-1, BDP-2, BDP-3 (10 μM) in THF.

**Fig. 2:**
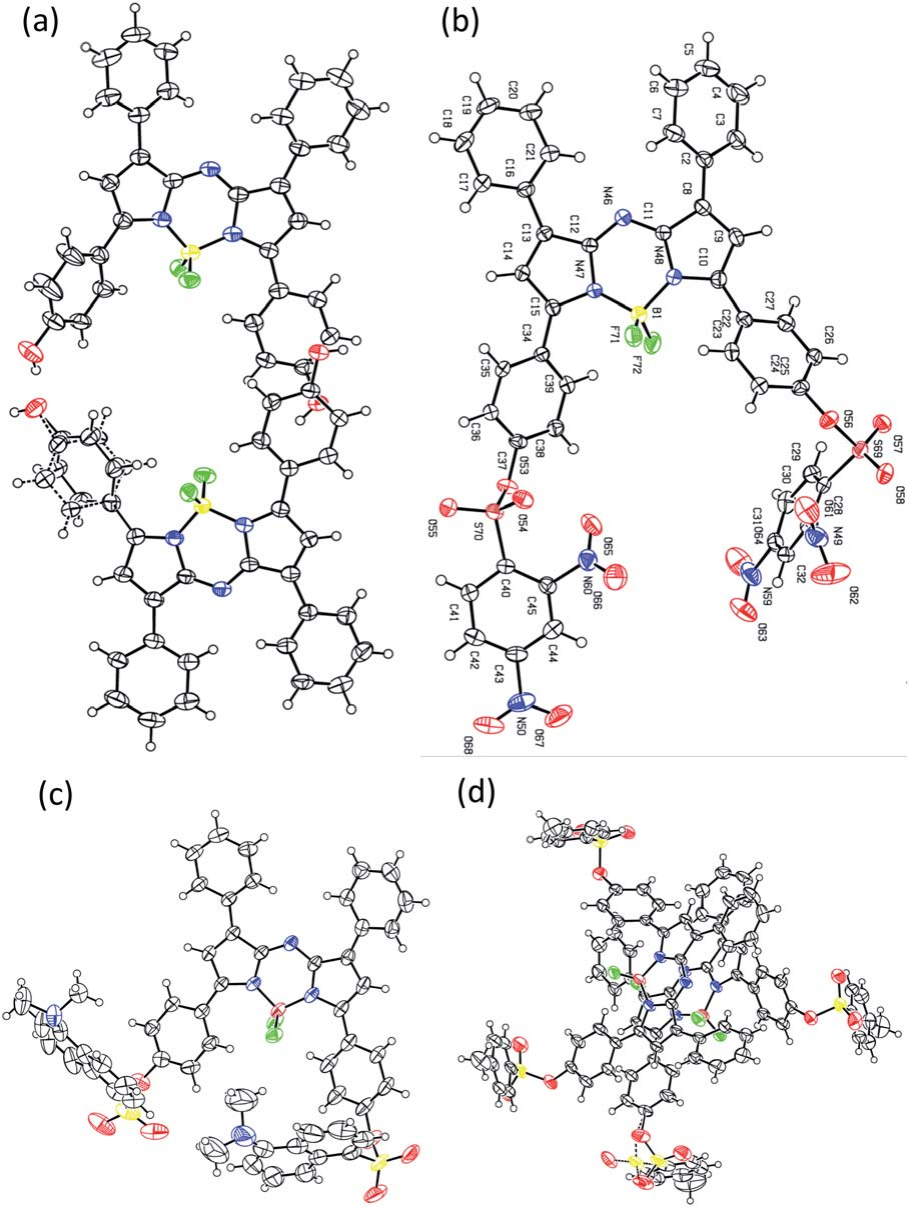
X-ray crystal structures of (a) BDP-OH, (b) BDP-1, (c) BDP-2, and (d) BDP-3.

BDP-OH was synthesized as described in the literature (Scheme S1†).^20,21^ The synthetic route of aza-BODIPY derivatives is depicted in Fig. 1a. The reaction of BDP-OH and several sulfonyl chloride compounds in the presence of TEA afforded BDP-1, BDP-2 and BDP-3 as dark-blue solids with an approximately 75% yield following extraction with H_2_O and drying over Na_2_SO_4_. The obtained structure was characterized using ^1^H NMR, ^13^C NMR, ESI HRMS and single crystal structure analysis (ESI†). Interestingly, the crystal structure of BDP-OH was obtained in dimer form, which showed the hydro interaction of –OH groups between two molecules (Fig. 2a and S17a†). Specifically, the crystal data of BDP-3 (Fig. 2d and S17d†) exhibited alternate arrangement between two molecules *via* π–π stacking of aromatic rings and intermolecular electrostatic interactions of B and F atoms.

**Fig. 3:**
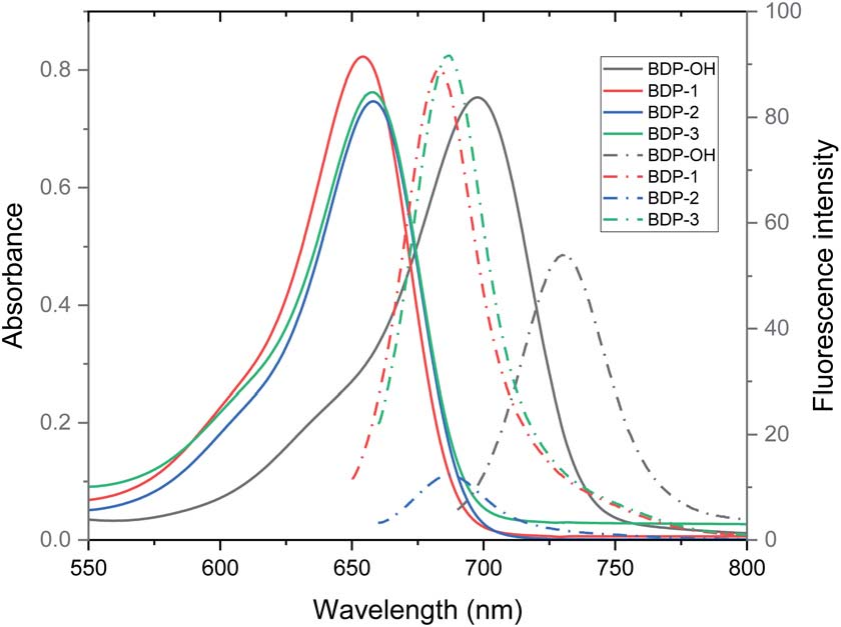
UV/vis (solid line) and fluorescence emission (dash dot line) spectra of BDP-OH (*λ*_ex_ = 685 nm), BDP-1, BDP-2, and BDP-3 (*λ*_ex_ = 645 nm) (10 μM) in THF (slit 5/5 nm).

As shown in Fig. 3, BDP-OH shows a broad absorption band from 600 nm to 750 nm. The central peak located at 697.8 nm is assigned from the S_0_ → S_1_ electron transition and corresponds to green color in THF solution (Fig. 1b). Furthermore, a shoulder absorption around 600 to 680 nm originated in the S_0_ → S_3_ electron transition of BDP-OH (Fig. S27†). BDP-1, BDP-2, and BDP-3 exhibit approximately 40 nm blue-shift absorption compared to BDP-OH, which is attributed to the introduction of sulfonyl chloride groups (electron withdrawing groups, EWG) in the hydroxyl positions of BDP-OH. The sulfonyl-aza-BODIPY conjugates display a broad absorption band around 550 to 700 nm, and a central peak around 657 nm corresponding to their dark-green color in THF solution attributed to the S_0_ → S_9_, S_0_ → S_3_ and S_0_ → S_1_ electron transition of BDP-1, BDP-2, and BDP-3, respectively (Fig. S25†). In addition, a shoulder absorption around 560–640 nm originated subsequently from the S_0_ → 2-singlet excited states of sulfonyl-aza-BODIPY (Fig. S25†). The molar absorbance coefficients of all aza-BODIPY derivatives are relatively high (7.4–8.5 × 10^5^ L mol^−1^ cm^−1^) in THF, with a slight decrease in other solvents such as MC, DMSO, and ACN (Table S3†). BDP-OH exhibits the emission peak at 731 nm with a fluorescence quantum yield (*Φ*_F_) of 2.58% due to strong ICT effect, which originated electron transfer from two –OH groups to aza-BODIPY core.^22–24^ The BDP-1–BDP-3 exhibits an emission peak at about 685 nm with a *Φ*_F_ value of 0.46–3.30% (in THF) owing to emission inhibition from strong sulfonyl acceptors (Table 1). These fluorescence quantum yields were significantly lower than that of aza-BODIPY core (*Φ*_F_ = 34%).^25,26^ In addition, their emission is decreased in DMSO and ACN due to fluorescence quenching in aggregate state.

**Table 1:** Photophysical and photosensitizing properties of BDP-OH, BDP-1–BDP-3 (in THF)

	*λ*_abs_ (nm)	*ε* (M^−1^ cm^−1^)	*λ*_ems_ (nm)	*Δ*_v_ (nm)	*Φ*_F_ (%)
BDP-OH	697.8	75 408	731.0	33.2	2.58
BDP-1	654.2	82 280	682.6	28.4	3.05
BDP-2	658.2	74 746	686.2	28.0	0.46
BDP-3	657.8	76 297	686.8	29.0	3.30

**Fig. 4:**
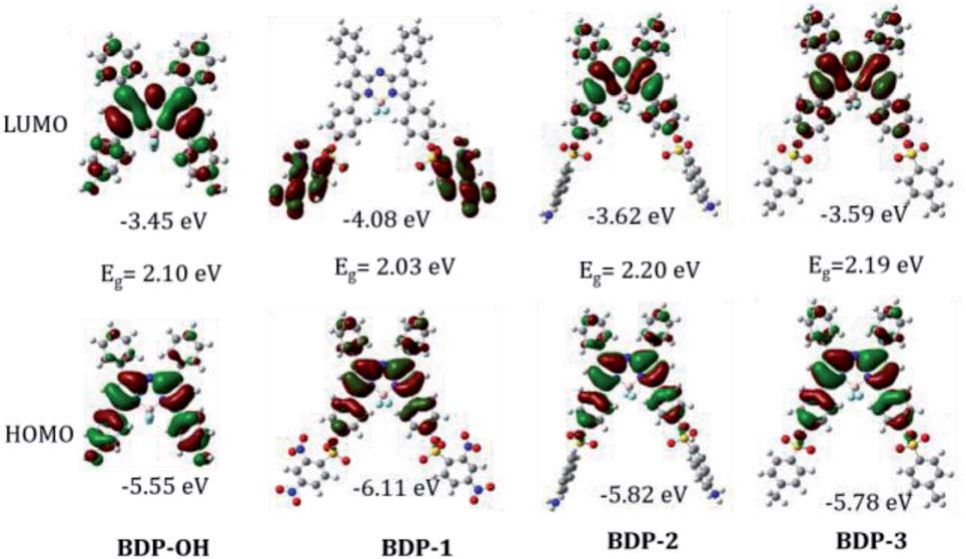
Molecular orbitals and energies (eV) of BDP-OH, BDP-1, BDP-2, and BDP-3 in the ground state (S_0_) obtained from the DFT calculations.

The HOMO of all aza-BODIPY derivatives is probably localized in the BODIPY core and the phenyl ring at 3-/5-position. However, the LUMO of BDP-1 is concentrated in 2,4-dinitrobenzenesulfonyl (DNBS) subunits, whereas the LUMO of BDP-OH, BDP-2 and BDP-3 is mostly located in the BODIPY core (Fig. 4). Furthermore, the HOMO–LUMO energy gap *E*_g_ of BDP-1 is lower than that of BDP-2 and BDP-3. Therefore, these specific phenomena are induced by DNBS moiety, which is known as an electron sink^27^ and a stronger EWG compared to 5-(dimethylamino)naphthalene-1-sulfonyl and toluene sulfonyl. Natural transition-orbital (NTO) analysis was performed to visualize the nature of different molecular excited states (Fig. S26†).^28^ Although, the contribution to the central peak and shoulder absorption band by different orbitals from HOMO−4 to LUMO+2 varies (Table S5†), NTOs indicate the uniformity of electronic transition in aza-BODIPY framework, which corresponds to similar UV/vis absorption spectra and naked-eye color of BDP-1–BDP-3.

**Fig. 5:**
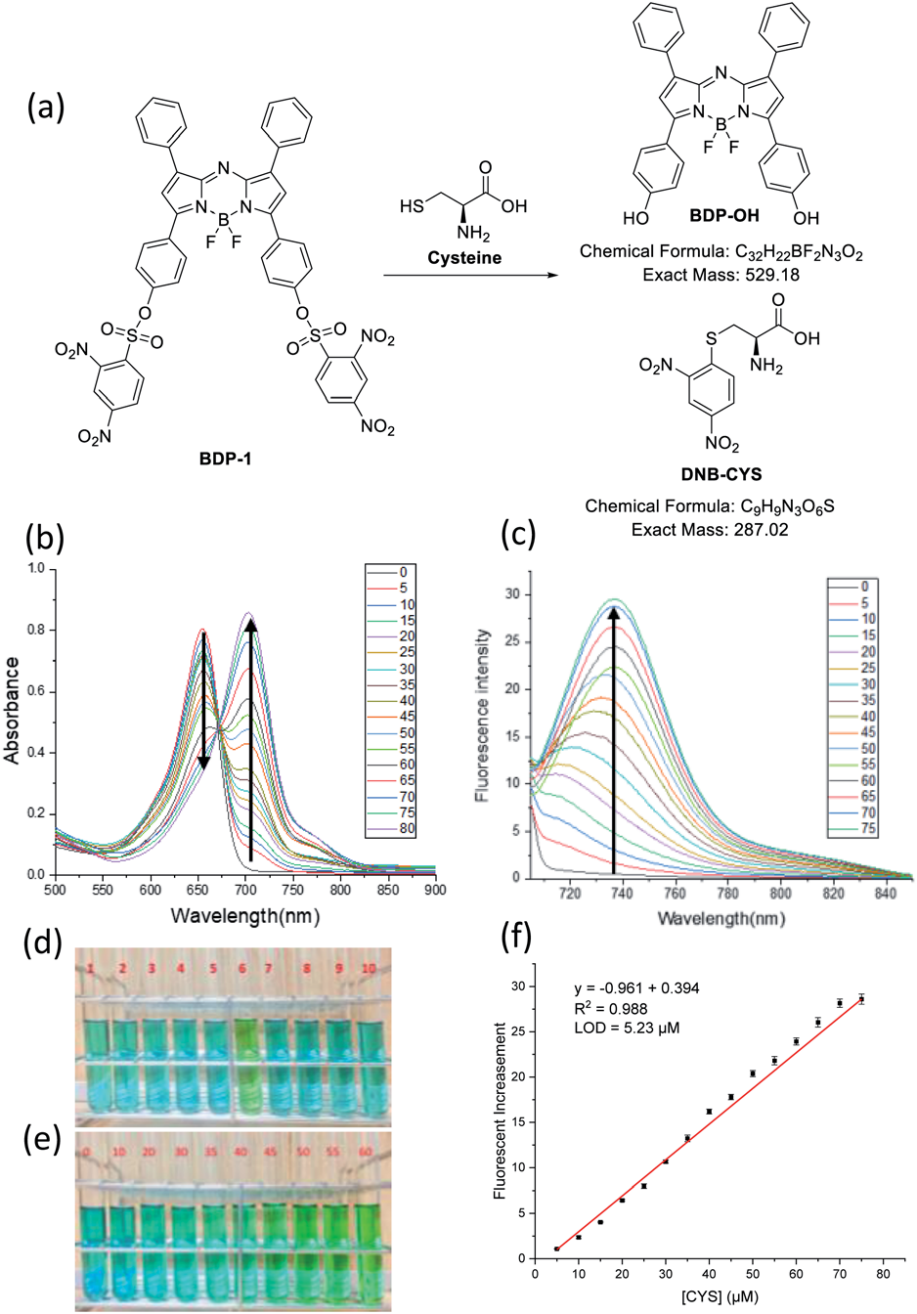
(a) The reaction of BDP-1 and Cys. (b) UV/vis and (c) fluorescence emission spectra titrations of BDP-1 (10 μM) in PBS (pH 7.4)/THF (5/5) (*λ*_ex_ = 700 nm) in the presence of Cys (0–80 μM) (slit 5/5 nm). (d) Color change of BDP-1 (10 μM) in the presence of (1) blank, (2) H_2_S (200 μM), (3) 2-mercaptoethanol (200 μM), (4) thioglycolic acid (200 μM), (5) GSH (1 mM), (6) Cys (50 μM), (7) Hcy (1 mM), (8) Na_2_S_2_O_7_ (200 μM), (9) KSCN (200 μM), (10) cysteamine (500 μM) in THF/PBS pH 7.4 (5/5). (e) Color change of BDP-1 (10 μM) in the presence of Cys at a concentration ranging from 0–60 μM in PBS (pH 7.4)/THF (5/5). (f) Plot of fluorescence intensity of BDP-1 as a function of Cys concentration in PBS (pH 7.4)/THF (5/5). The visualize and spectra results were recorded during 3 min.

**Fig. 6:**
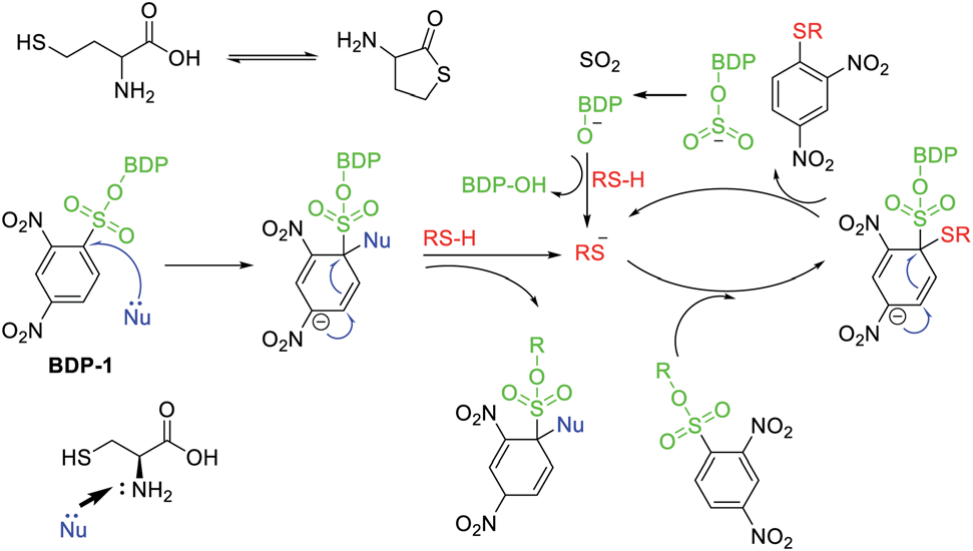
Proposal mechanism for reaction of Cys and BDP-1.

The recognition of several thiols by BDP-1, BDP-2, and BDP-3 was investigated *via* colorimetric changes, and changes in UV absorption and fluorescence emission in pH 7.4 PBS buffer/THF (5/5) (Fig. 5 and S20–S22†). We investigated the colorimetric responses of BDP-1, BDP-2 and BDP-3 (10 μM) with H_2_S, 2-mercaptoethanol, thioglycolic acid, glutathione, cysteine (Cys), and homocysteine (Hcy), Na_2_S_2_O_7_, KSCN, cysteamine at different concentrations (50–1000 μM). Only Cys induced dark green to green color transition toward BDP-1 at 50 μM during 3 min, whereas no changes were detected with other reagents at higher concentrations or longer incubated time (Fig. 5d), demonstrated that Cys efficiently reacted with BDP-1. Furthermore, the UV-vis and fluorescence spectra of BDP-1 in the high presence of Hcy or GSH (1 mM) was slightly change, indicated that Hcy and GSH reacted with BDP-1. The color of BDP-2 and BDP-3 solution was not altered by the addition of all reagents at concentrations as high as 1 mM during more than 30 min. The reaction of biothiol with sulfonyl groups based on nucleophilic aromatic substitution mechanism and the size of thiol structures (Fig. 6). The electron withdrawing ability of 2,4-dinitrobenzene is significantly stronger than that of toluene or (dimethylamino)naphthalene, so only BDP-1 can react to Cys, Hcy or GSH, whereas BDP-2 and BDP-3 cannot do that. On the other hand, the free –SH group plays an important role in the reaction (Fig. 6) of biothiol with BDP-1. It has known that Hcy can cyclize to give homocysteine thiolactone, a five-membered heterocycle,^29^ so Hcy barely react with BDP-1 compared to Cys even though these are small molecules. Thus, only UV-vis and fluorescence emission spectra of BDP-1 were slightly changed (Fig. S20†), but the color of BDP-1 was unchanged in the high-concentration of Hcy (1 mM).^30–32^

The color change of BDP-1 in the presence of Cys was confirmed by UV/vis absorption and fluorescence emission spectra (Fig. S20 and S23†). Upon Cys addition, the peak of BDP-1 centered on the absorption band shifted from 655 nm to 700 nm together with fluorescence turn-on about 730 nm (*λ*_ex_ = 700 nm), which similar to the absorption and emission spectra of BDP-OH. On the other hand, the fluorescence emission spectra titrations of BDP-1 at 675 nm excitation in the presence of cysteine were investigated. In which, the fluorescence emission at 685 nm gradually decreased, which was originated from the disappearance of BDP-1. It indicates that BDP-1 reacts with Cys to form BDP-OH and DNB-Cys (Fig. 5a). UV/vis absorption and fluorescence emission spectra of BDP-2 and BDP-3 were mostly unchanged under the high concentrations of GSH, Cys and Hcy, corresponding to their color. These results indicate the sensitivity and selectivity of BDP-1 toward Cys. In addition, ESI HRMS peaks *m*/*z* = 528.1707 [M − H]^−^, calc. for C_32_H_21_BF_2_N_3_O_2_ (BDP-OH) and *m*/*z* = 286.0145 [M + H]^−^, calc. for C_9_H_9_N_3_O_6_S (DNB-Cys) (Fig. S16†) confirm the reaction of BDP-1 and Cys. Analysis of changes in UV/vis absorption spectra of BDP-1 (10 μM) in PBS buffer (pH 7.4)/THF (5/5) suggests that the original 655 nm absorption band of BDP-1 decreased together with a simultaneous increase at 700 nm upon binding with Cys (Fig. 5b). As shown in Fig. 5c, the emission intensity of BDP-1 was gradually enhanced by increasing Cys levels from 0 to 80 μM. The limit of detection (LOD) of BDP-1 for Cys was calculated at 5.23 μM (Fig. 5f). In addition, the colorimetric response value (CR) of BDP-1 (10 μM) was observed at 56.99% in the presence of 50 μM Cys (Fig. S23b†).

## Experimental

### Synthesis

Synthesis of BDP-1–BDP-3:^11,33^ sulfonyl chloride compound (1.2 mmol) was diluted in dichloromethane (10 mL) at 0 °C. The mixture was slowly added a solution of BDP-OH (0.4 mmol), TEA (0.2 mL) in dichloromethane (20 mL) and then was stirred at room temperature overnight. The solution was extracted with H_2_O, dried over Na_2_SO_4_, and concentrated to give a black-blue solid as product (yield about 75%).

#### BDP-1

^1^H NMR (400 MHz, acetone-*d*_6_) *δ* 9.02 (d, *J* = 2.2 Hz, 2H), 8.74 (dd, *J* = 8.7, 2.2 Hz, 2H), 8.44 (d, *J* = 8.7 Hz, 2H), 8.21 (t, *J* = 7.3 Hz, 8H), 7.58–7.49 (m, 8H), 7.46–7.42 (m, 4H); ^13^C NMR (101 MHz, acetone-*d*_6_) *δ* 205.37, 158.09, 151.93, 150.81, 148.96, 145.81, 144.79, 134.03, 132.29, 132.00, 131.31, 130.11, 129.57, 128.87, 127.38, 122.48, 121.15, 120.18; ESI HRMS *m*/*z* = 989.10 [M]^+^, calc. for C_44_H_26_BF_2_N_7_O_14_S_2_ = 989.10.

#### BDP-2

^1^H NMR (400 MHz, chloroform-*d*) *δ* 8.60 (dt, *J* = 8.6, 1.1 Hz, 2H), 8.46 (dt, *J* = 8.7, 0.9 Hz, 2H), 8.10 (dd, *J* = 7.4, 1.3 Hz, 2H), 7.99–7.90 (m, 4H), 7.86–7.78 (m, 4H), 7.68 (dd, *J* = 8.7, 7.6 Hz, 2H), 7.46–7.36 (m, 8H), 7.27–7.24 (m, 2H), 7.03–6.95 (m, 4H), 6.87 (d, *J* = 1.2 Hz, 2H), 2.90 (s, 12H); ^13^C NMR (101 MHz, chloroform-*d*) *δ* 207.16, 158.04, 152.13, 151.53, 145.72, 144.57, 132.39, 132.04, 131.44, 131.21, 130.96, 130.20, 130.12, 129.90, 129.86, 129.45, 129.32, 128.77, 123.10, 122.41, 119.42, 119.11, 115.84, 45.56; ESI HRMS *m*/*z* = 996.2878 [M + H]^+^, calc. for C_56_H_44_BF_2_N_5_O_6_S_2_ = 995.28.

#### BDP-3

^1^H NMR (400 MHz, chloroform-*d*) *δ* 8.07–7.99 (m, 4H), 7.99–7.92 (m, 4H), 7.78–7.69 (m, 4H), 7.51–7.37 (m, 6H), 7.37–7.26 (m, 4H), 7.14–7.04 (m, 4H), 6.98 (d, *J* = 1.3 Hz, 2H), 2.43 (s, 6H); ^13^C NMR (101 MHz, chloroform-*d*) *δ* 158.16, 151.47, 145.81, 132.36, 132.09, 131.27, 130.32, 130.01, 129.93, 129.50, 128.83, 128.63, 122.73, 119.17, 21.85; ESI HRMS *m*/*z* = 860.1852 [M + Na]^+^, calc. for C_46_H_33_BF_2_N_3_O_6_S_2_ = 837.20.

### Computational data

The DFT calculations of the aza-BODIPY derivatives were performed using the Gaussian 09 program package. These geometric structures were optimized without imaginary frequencies using the B3LYP hybrid function together with the 6-31+G(d) basic sets for C, H, B, O, and F atoms; 6-31+G(2d,p) basic sets for S and N atoms.^34^ Several important bond lengths and angles of these optimized structures are highly similar to those of crystal structures obtained.

Optical excitation energies were calculated with various functional parameters using time-dependent DFT (TD-DFT) comprising the hybrid, gradient-corrected, popular local, and functional methods. The 6-31G(d) basic sets were used for C, H, B, and O, F while 6-31G(2d,p) basic sets were used for S and N in THF solvent in the polarizable continuum model (PCM) using the integral equation formalism variant (IEFPCM).^35^ Among various functionals, LSDA functional showed that the calculated excitation energies were closer to the experimental data with mean absolute deviation (MAD) as low as 0.04 (Table S4†). This finding will facilitate current and future TD-DFT calculations about (aza-)BODIPY and/or sulfonyl chloride structures.

## Conclusions

We have synthesized, crystallized and studied photophysical properties of several near-infrared fluorescent sulfonyl aza-BODIPY. Among these structures, BDP-1 was prepared by introducing 2,4-dinitro-1-sulfonyl chloride to BDP-OH. Upon treatment with Cys, the BDP-1 displayed a selective colorimetric shift from dark-green to green, as well as red-shift enhancement of fluorescence. The sulfonyl group of BDP-1 efficiently reacted with Cys, resulting in the formation of BDP-OH and DNB-Cys. BDP-1 showed a high selectivity for Cys among other biothiols due to nucleophilic aromatic substitution mechanism and small size of Cys. The HOMO–LUMO energy gap was also calculated *via* TD-DFT calculation and the value matched UV/vis and fluorescence emission spectra precisely. The calculated detection limit of BDP-1 was 5.23 μM and the colorimetric response (CR) value was 56.99%. Finally, the reaction between BDP-1 and Cys was confirmed *via* mass spectroscopy.

## Author contributions

Thanh Chung Pham conceived and designed the probes and calculated the DFT in the Gaussian 09 package, Yeonghwan Choi performed and collected the UV-vis spectra and fluorescence spectra, Chaeeon Bae performed and collected the UV-vis spectra and fluorescence spectra, Cong So Tran synthesized aza-BODIPY derivatives, Dongwon Kim obtained single crystal data, Ok-Sang Jung interpreted final crystal structures, Yong-Cheol Kang performed the fluorescence spectra and calculated quantum yield, SungYong Seo studied ^1^H NMR, ^13^C NMR, HRMS, Hyun Sung Kim performed computational study, Hwayoung Yun designed the scheme to synthesize probes, Xin Zhou designed aza-BODIPY derivatives for NIR probes, Songyi Lee designed BDP-OH, BDP-1, BDP-2, BDP-3 and wrote the paper.

## Conflicts of interest

There are no conflicts to declare.

## Supplementary Material

RA-011-D0RA10567H-s001

RA-011-D0RA10567H-s002
