# Supplementary material for: A molecular design towards sulfonyl aza-BODIPY based NIR fluorescent and colorimetric probe for selective cysteine detection
Source: RSC Adv. 2021 Mar 10;11(17):10154–8. doi: 10.1039/d0ra10567h (PMC8695679; doi:10.1039/d0ra10567h)
Supplement: RA-011-D0RA10567H-s001 [file RA-011-D0RA10567H-s001.pdf]

Electronic Supplementary Material (ESI) for RSC Advances.
This journal is © The Royal Society of Chemistry 2021

Supporting Information

A molecular design towards Sulfonyl aza-BODIPY based NIR fluorescent and colorimetric probe for selective cysteine detection

Thanh Chung Pham,[b] Yeonghwan Choi,[b] Chaeeon Bae,[b] Cong So Tran,[c] Dongwon Kim,[d] Ok-Sang Jung,[d] Yong-Cheol Kang,[a] SungYong Seo,[a] Hyun Sung Kim,[a] Hwayoung Yun,*[c] Xin Zhou*[e] and Songyi Lee*[a,b]

[a]*Department of Chemistry, Pukyong National University, Busan 48513, Korea*
[b]*Industry 4.0 Convergence Bionics Engineering, Pukyong National University, Busan 48513, Korea*
[c]*College of Pharmacy, Pusan National University, Busan 46241, Korea*
[d]*Department of Chemistry, Pusan National University, Busan 46241, Korea*
[e]*Department of Chemistry, Qingdao University, Qingdao 266071, P. R. China*

*Corresponding author: hyun@pusan.ac.kr; zhouxin@qdu.edu.cn; slee@pknu.ac.kr

1. Experimental data 2
1.1. General consideration 2
1.2. Synthetic process 2
1.3. Photophysical measurements 3
2. Results 4
2.1. NMR and Mass spectra 4
2.2. Crystal data 10
2.3. Photophysical results 12
2.4. DFT calculation results 17

1. Experimental data

1.1. General consideration

All reagents and organic solvents used in the synthesis were obtained from Aldrich, TCI (South Korea) and used without further purification. Flash chromatography was carried out on silica gel (230-400 mesh) followed by determination of ^{1}H and ^{13}C NMR spectra using a Bruker Avance 400 MHz spectrometer. Mass spectra were obtained using a maXis-HD (Bruker). UV absorption spectroscopy measurements were carried out on V-730 UV-Visible Spectrophotometer (Jasco) at room temperature. Fluorescence emission spectra were obtained using an F-7000 Fluorescence Spectrophotometer (Hitachi High-Tech).

1.2. Synthetic process

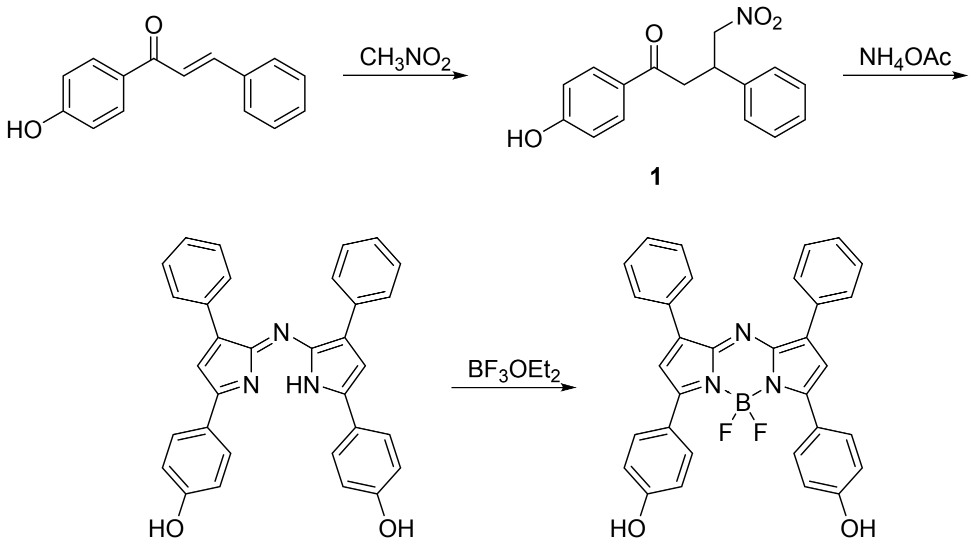

Scheme S1. Synthesis of **BDP-OH** and sulfonyl aza-BODIPY.

Synthesis of **2:** The mixture of **1** (2.0 g, 7.01 mmol), ammonium acetate (10 g, 130 mmol) in ethanol (50 mL) was refluxed during 24h. The solution was cooled down to 0 °C and the blue solid residue was filtered as product (yield 35.3 %). ^{1}H NMR (400 MHz, DMSO-d_6) δ 10.28 (s, 1H), 8.12 - 8.04 (m, 4H), 7.97 - 7.89 (m, 4H), 7.54 (s, 2H), 7.50 - 7.41 (m, 4H), 7.41 - 7.33 (m, 2H), 7.05 - 6.97 (m, 4H). ^{13}C NMR (101 MHz, DMSO-d_6) δ 160.60, 154.88, 149.03, 133.99, 129.10, 128.85, 128.50, 123.00, 116.98, 115.61. ESI HRMS m/z = 482.1870 $[M+H]^+$, calc. for $C_{32}H_{23}N_3O_2$ = 481.18.

Synthesis of **BDP-OH:** The solution of **2** (600 mg, 1.25 mmol) in anhydrous DCM (30mL) was added i-Pr_2EtN (5.0 mL, 16.8 mmol), and then was stirred for 15 min at room

temperature. $BF_3 \cdot OEt_2$ (5.0 mL, 23.8 mmol) was slowly added and the obtained mixture was stirred overnight at room temperature. The solution was washed with H_2O, dried over Na_2SO_4, and concentrated to give a black-blue solid as product (yield 95.2 %). ^1H NMR (400 MHz, DMSO-d_6) δ 10.49 (s, 2H), 8.19 - 8.12 (m, 4H), 8.12 - 8.06 (m, 4H), 7.59 - 7.49 (m, 6H), 7.49 - 7.42 (m, 2H), 6.99 - 6.90 (m, 4H). ^{13}C NMR (101 MHz, DMSO-d_6) δ 161.47, 157.89, 144.85, 142.11, 132.63, 132.47, 129.95, 129.58, 129.22, 122.26, 120.05, 116.44. ESI HRMS m/z = 552.1665 $[M+Na]^+$, calc. for $C_{32}H_{22}BF_2N_3O_2$ = 529.18.

1.3. Photophysical measurements

Fluorescence QY (Φ_F): Dilute sample solutions (typically 10 μM) were prepared from stock solutions (1.0 mM) at ambient conditions. The UV/Vis and fluorescence spectra of solutions (10 μM) were obtained using a 1 cm quartz cuvette. The fluorescence QY (Φ_F) of the aza-BODIPY derivatives was determined according to following equation:

$$\Phi_s = \Phi_{ref} \times \frac{I_s}{I_{ref}} \times \frac{A_{ref}}{A_s} \times \left(\frac{\eta_s}{\eta_{ref}}\right)^2$$

where Φ_{ref} is the Φ_F of reference, I is the area under the emission spectra, A is the absorbance at the excitation wavelength, and η is the refractive index of the used solvent. s and ref stand for aza-BODIPY derivatives and reference, respectively. Rhodamine 6G (Φ_{ref} = 0.94 in ethanol) was used as a reference for the fluorescence QY.

The limit of detection (LOD) was estimated via using the following equation:

$$LOD = 3 \times \delta \div S$$

where δ represents the standard deviation of the blank measurements, and S is the slope of the intensity versus sample concentration curve (Figure 6c).

The CR (colorimetric response) (%) was calculated using the following equation:

$$CR = \frac{PB_0 - PB_1}{PB_0} \times 100$$

where PB= $A_{dark\text{-}green}/(A_{dark\text{-}green} + A_{green})$, A denotes the absorbance at either the dark-green or the green component in the UV/Vis spectrum.

2. Results

2.1. NMR and Mass spectra

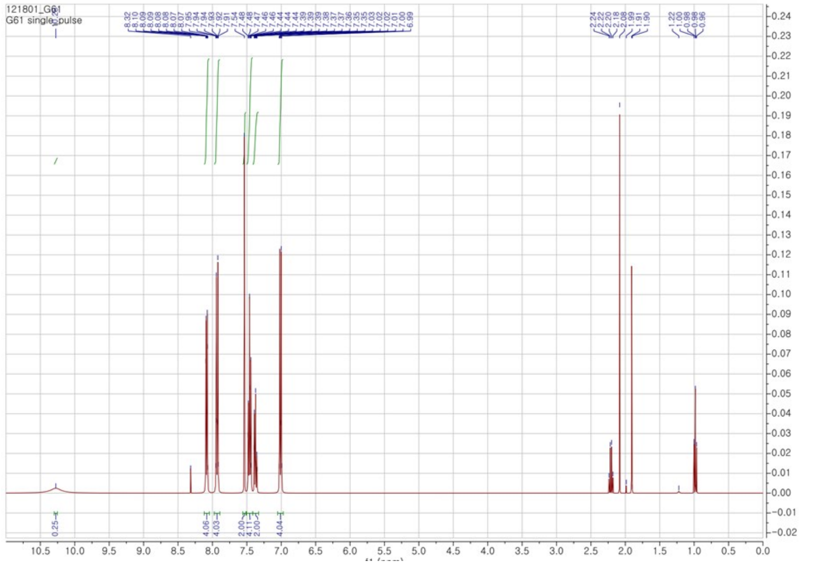

Figure S1. ^{1}H NMR spectrum of **2** in DMSO-d_6.

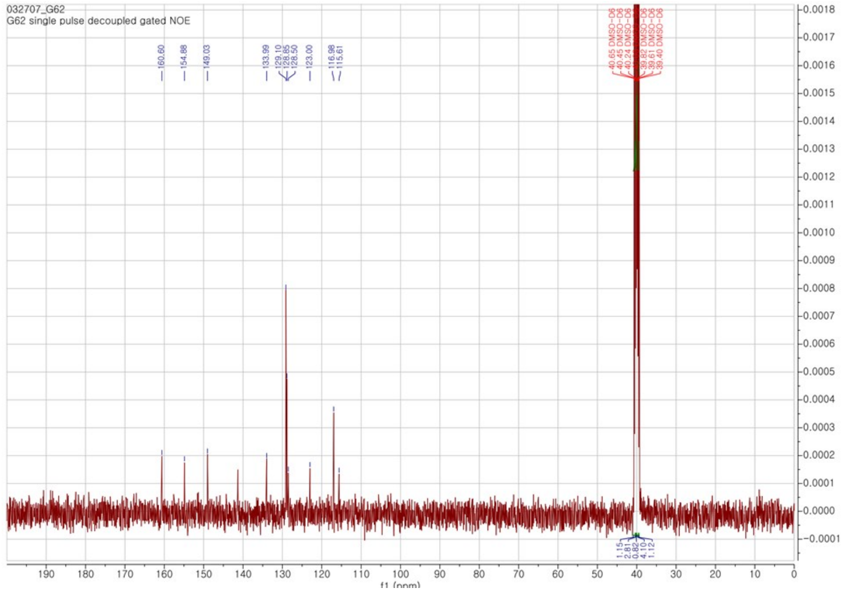

Figure S2. ^{13}C NMR spectrum of **2** in DMSO-d_6.

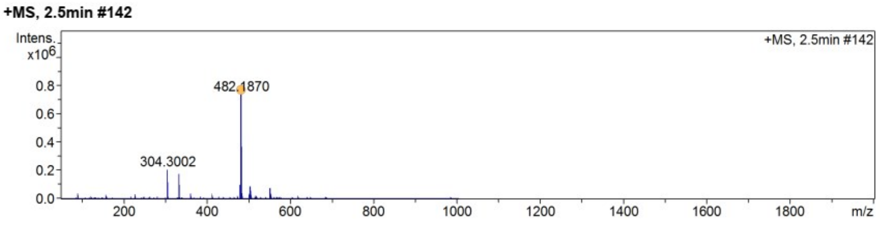

Meas. m/z	#	Ion Formula	m/z	err [ppm]	mSigma	# mSigma	Score	rdb	e⁻ Conf	N-Rule
482.1870	1	C32H24N3O2	482.1863	-1.5	15.8	1	100.00	22.5	even	ok

Figure S3. Mass spectrum (HRMS) of **2**

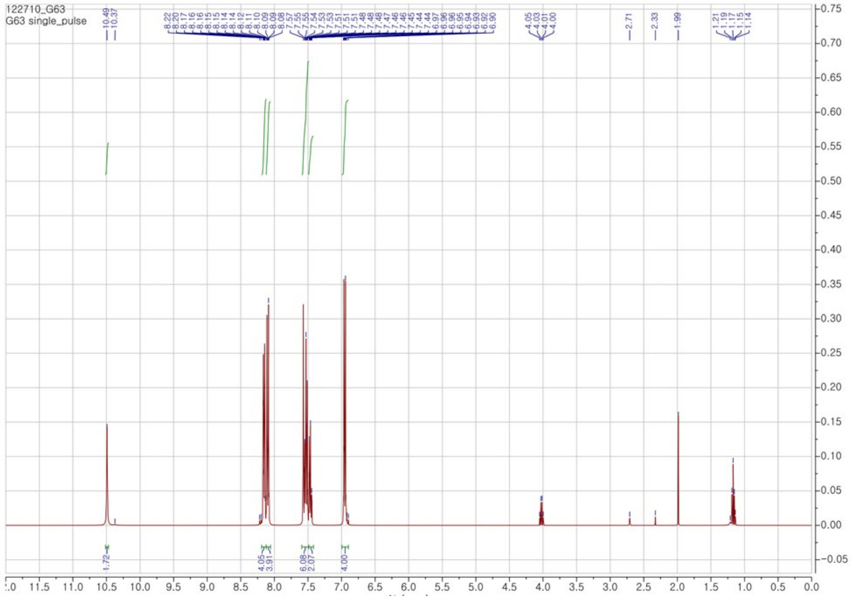

Figure S4. ^{1}H NMR spectrum of **BDP-OH** in DMSO-d_6.

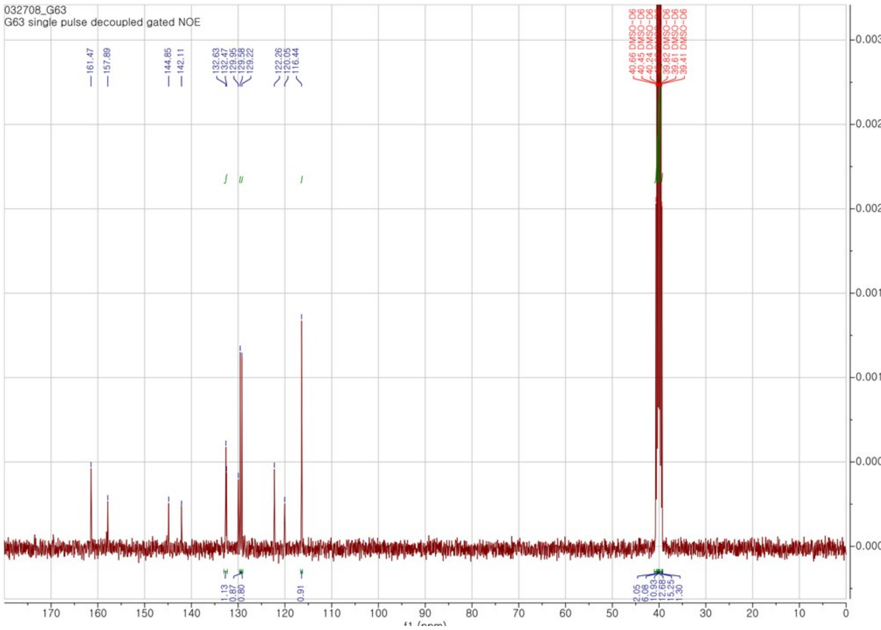

Figure S5. ^{13}C NMR spectrum of **BDP-OH** in DMSO-d_6.

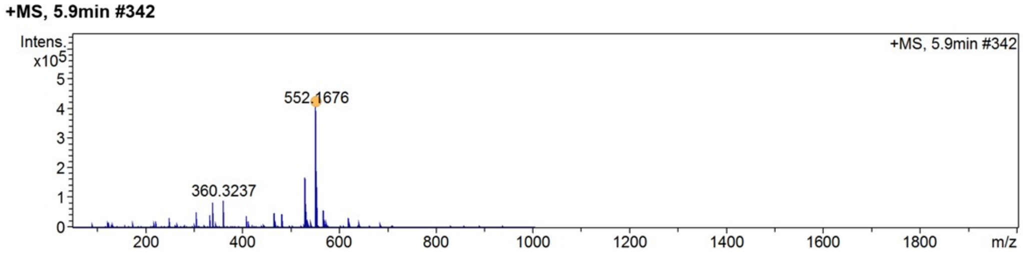

Meas. m/z	#	Ion Formula	m/z	err [ppm]	mSigma	# mSigma	Score	rdb	e⁻ Conf	N-Rule
552.1676	1	C32H22BF2N3NaO2	552.1665	-0.9	1.7	1	100.00	22.5	even	ok

Figure S6. Mass spectrum (HRMS) of **BDP-OH**

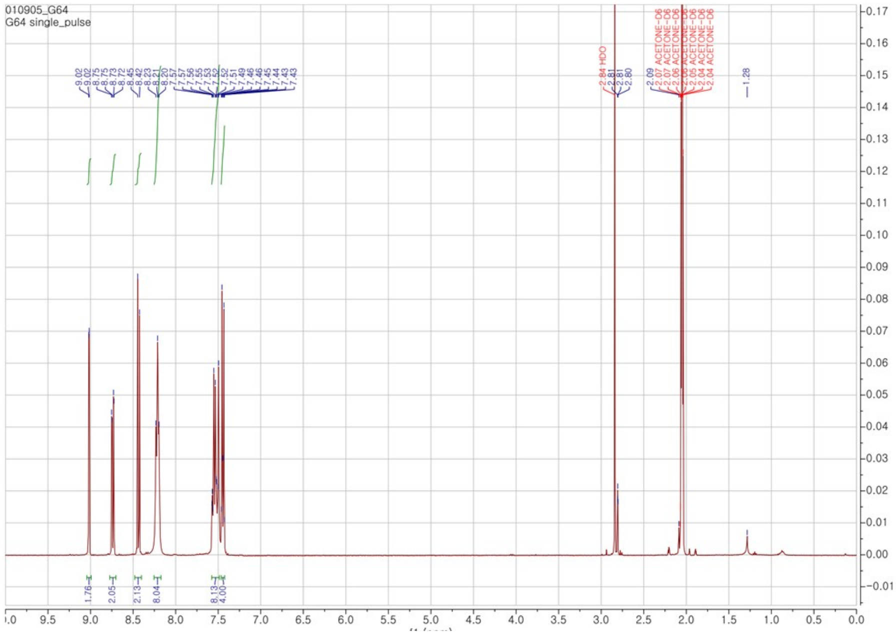

Figure S7. ^{1}H NMR spectrum of **BDP-1** in *Acetone-d_6*.

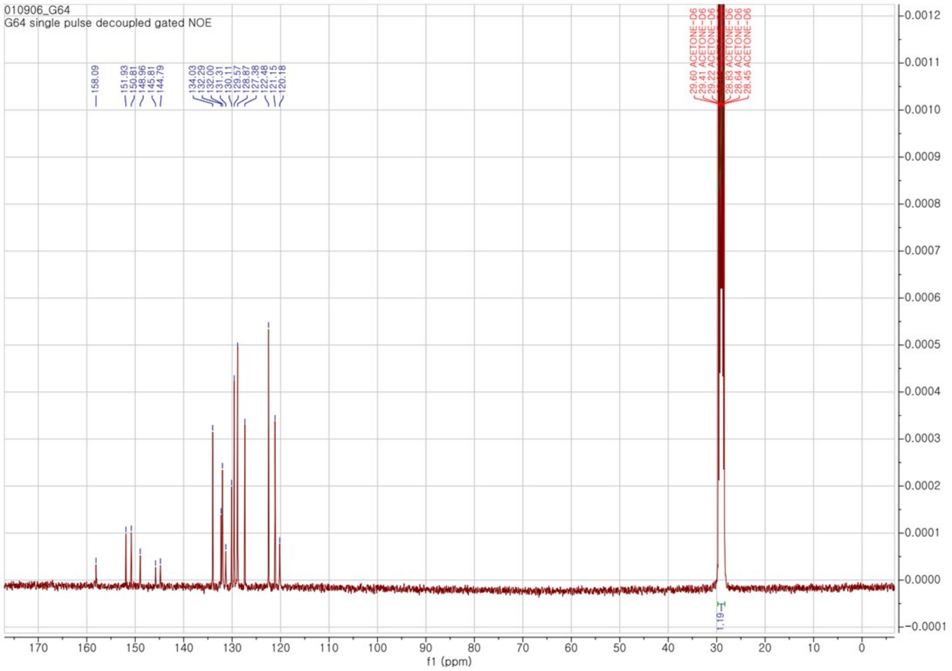

Figure S8. ^{13}C NMR spectrum of **BDP-1** in Acetone-d_6.

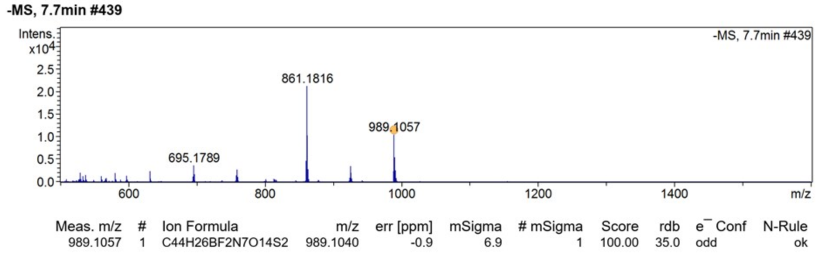

Figure S9. Mass spectrum (HRMS) of **BDP-1**

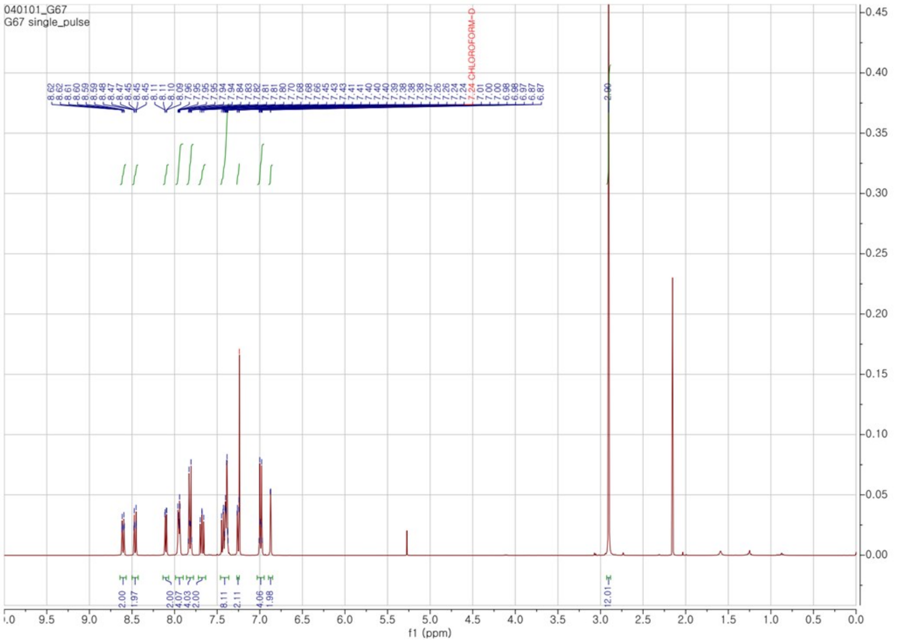

Figure S10. ^{1}H NMR spectrum of **BDP-2** in $CDCl_3$

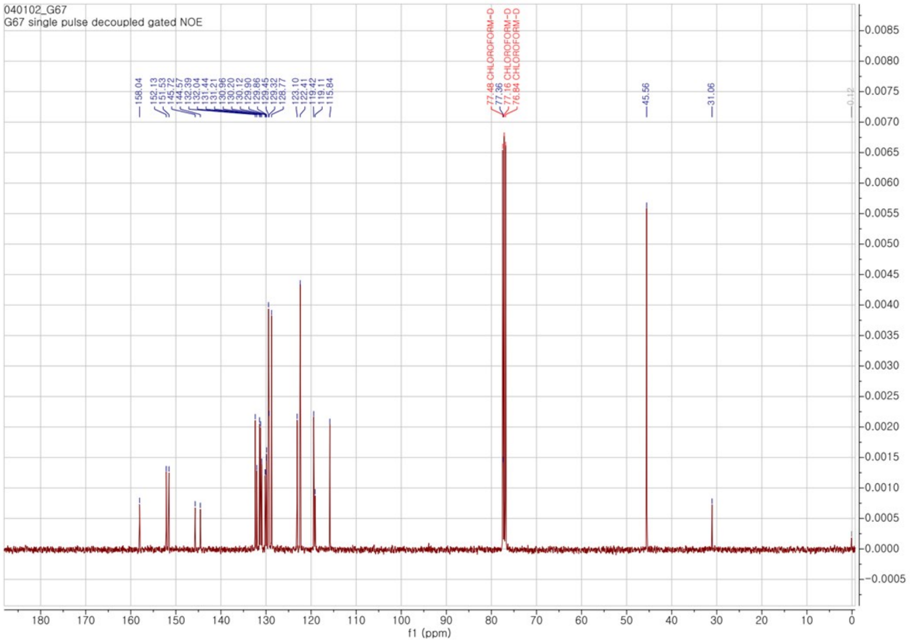

Figure S11. ^{13}C NMR spectrum of **BDP-2** in $CDCl_3$

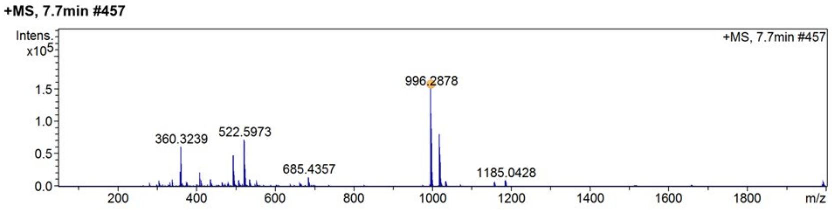

Meas. m/z	#	Ion Formula	m/z	err [ppm]	mSigma	# mSigma	Score	rdb	e⁻ Conf	N-Rule
996.2878	1	C56H45BF2N5O6S2	996.2867	-0.2	19.5	1	100.00	36.5	even	ok

Figure S12. Mass spectrum (HRMS) of **BDP-2**

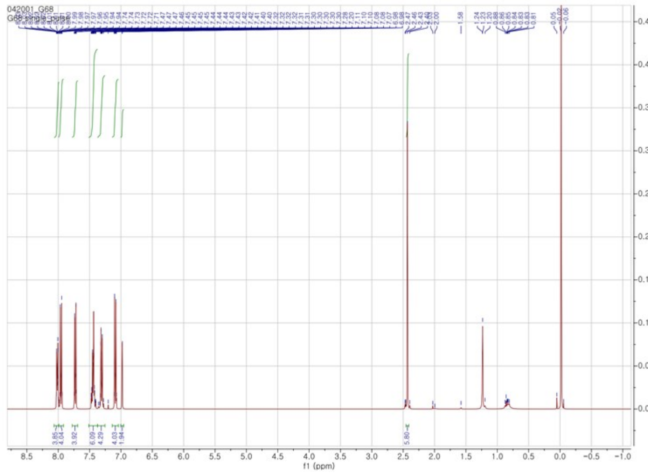

Figure S13. ^{1}H NMR spectrum of **BDP-3** in $CDCl_3$

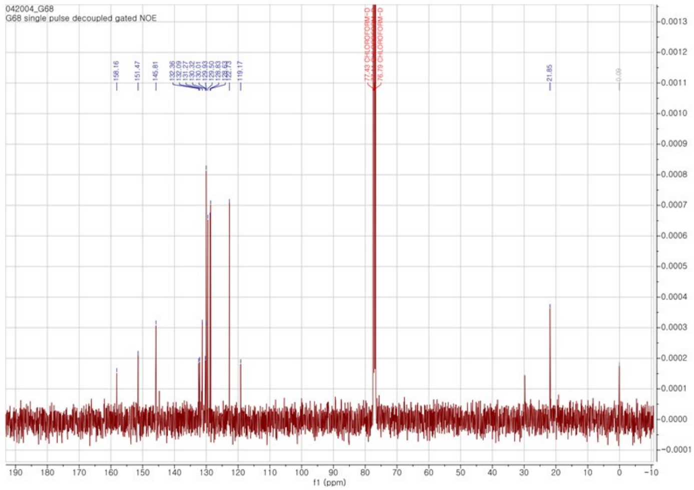

Figure S14. ^{13}C NMR spectrum of **BDP-3** in $CDCl_3$

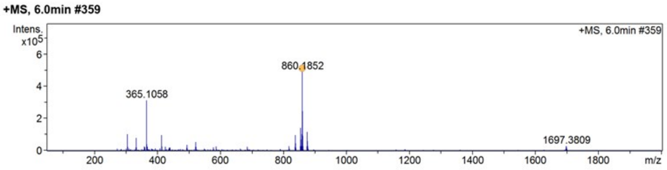

Meas. m/z	#	Ion Formula	m/z	err [ppm]	mSigma	# mSigma	Score	rdb	e⁻ Conf	N-Rule
860.1852	1	C46H34BF2N3NaO6S2	860.1842	-0.3	18.9	1	100.00	30.5	even	ok
	2	C48H36BF2NaO7S2	860.1856	1.3	23.8	2	55.73	30.0	odd	ok
	3	C47H30BF2N7NaO2S2	860.1856	1.3	24.1	3	56.23	35.5	even	ok
	4	C49H32BF2N4NaO3S2	860.1869	2.9	663.7	4	0.00	35.0	odd	ok

Figure S15. Mass spectrum (HRMS) of **BDP-3**

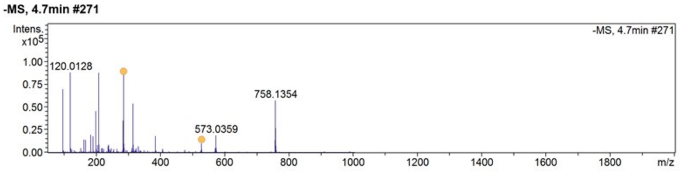

Meas. m/z	#	Ion Formula	m/z	err [ppm]	mSigma	# mSigma	Score	rdb	e⁻ Conf	N-Rule
286.0145	1	C9H8N3O6S	286.0139	-1.8	6.2	1	100.00	7.5	even	ok
528.1707	1	C32H21BF2N3O2	528.1700	-0.2	9.1	1	100.00	23.5	even	ok

Figure S16. Mass spectrum (HRMS) of **DNB-CYS** and **BDP-OH.**

2.2. Crystal data

Table S1. Crystal data of **BDP-OH** and **BDP-1**.

	**BDP-OH**	**BDP-1**
Empirical formula	$C_{32}H_{22}BF_2N_3O_2$	$C_{44}H_{26}BF_2N_7O_{14}S_2$
Formula weight	529.33	989.65
Temperature	225(2) K	193(2) K
Wavelength	0.700 Å	0.71073 Å
Crystal system	Orthorhombic	Monoclinic
Space group	$Pca2_1$	$P2_1/c$
Unit cell dimensions	a = 11.263(2) Å α= 90°.<br>b = 12.253(3) Å β= 90°.<br>c = 36.822(7) Å γ = 90°.	a = 17.300(4) Å α= 90°.<br>b = 26.810(6) Å β= 102.391(4)°.<br>c = 9.422(2) Å γ = 90°.
Volume	5081.6(18) Å^3	4268.3(17) Å^3
Z	8	4
Density (calculated)	1.384 Mg/m^3	1.540 Mg/m^3
Absorption coefficient	0.093 mm^{-1}	0.214 mm^{-1}
F(000)	2192	2024
Crystal size	0.120 x 0.083 x 0.063 mm^3	0.200 x 0.080 x 0.070 mm^3
Theta range for data collection	1.637 to 33.432°.	1.424 to 24.592°.
Index ranges	-17<=h<=17,-17<=k<=17,<br>-52<=l<=52	-20<=h<=20, -31<=k<=31, -<br>11<=l<=11
Reflections collected	53296	52201
Independent reflections	16873 [R(int) = 0.0710]	7149 [R(int) = 0.1133]
Completeness to theta = 24.835°	96.6 %	99.7 %
Absorption correction	Empirical	Semi-empirical from equivalents
Max. and min. transmission	1.000 and 0.865	0.7451 and 0.5830
Refinement method	Full-matrix least-squares on F^2	Full-matrix least-squares on F^2
Data / restraints / parameters	16873 / 13 / 785	7149 / 0 / 631
Goodness-of-fit on F^2	0.972	1.023
Final R indices [I>2sigma(I)]	R1 = 0.0537, wR2 = 0.1316	R1 = 0.0494, wR2 = 0.1007
R indices (all data)	R1 = 0.0864, wR2 = 0.1458	R1 = 0.0928, wR2 = 0.1204
Absolute structure parameter	0.5(2)	
Extinction coefficient	n/a	n/a
Largest diff. peak and hole	0.307 and -0.289 e.Å^{-3}	0.220 and -0.355 e.Å^{-3}
CCDC deposition number	2049235	2064541

Table S2. Crystal data of **BDP-2** and **BDP-3.**

	**BDP-2**	**BDP-3**
Empirical formula	$C_{56}H_{44}BF_2N_5O_6S_2$	$C_{46}H_{34}BF_2N_3O_6S_2$
Formula weight	995.89	837.69
Temperature	293(2) K	223(2) K
Wavelength	0.71073 Å	0.700 Å
Crystal system	Monoclinic	Triclinic
Space group	$P2_1/c$	P-1
Unit cell dimensions	a = 7.8870(16) Å α= 90°.<br>b = 42.104(8) Å β= 91.65(3)°.<br>c = 14.974(3) Å γ = 90°.	a = 16.040(3) Å α= 116.45(3)°.<br>b = 17.100(3) Å β= 103.07(3)°.<br>c = 17.864(4) Å γ = 103.99(3)°.
Volume	4970.4(17) $Å^3$	3929.8(18) $Å^3$
Z	4	4
Density (calculated)	1.331 Mg/m^3	1.416 Mg/m^3
Absorption coefficient	0.170 mm^{-1}	0.191 mm^{-1}
F(000)	2072	1736
Crystal size	0.075 x 0.060 x 0.040 mm^3	0.180 x 0.090 x 0.070 mm^3
Theta range for data collection	1.444 to 34.058°.	1.351 to 33.472°.
Index ranges	-11<=h<=11,-58<=k<=58,<br>-21<=l<=21	-25<=h<=25, -26<=k<=26,<br>-27<=l<=27
Reflections collected	57458	51986
Independent reflections	16997 [R(int) = 0.0716]	26475 [R(int) = 0.0347]
Completeness to theta = 24.835°	99.6 %	99.4 %
Absorption correction	Empirical	Empirical
Max. and min. transmission	1.000 and 0.975	1.000 and 0.909
Refinement method	Full-matrix least-squares on F^2	Full-matrix least-squares on F^2
Data / restraints / parameters	16997 / 0 / 653	26475 / 0 / 1122
Goodness-of-fit on F^2	0.974	1.135
Final R indices [I>2sigma(I)]	R1 = 0.0586, wR2 = 0.1599	R1 = 0.0942, wR2 = 0.2975
R indices (all data)	R1 = 0.1163, wR2 = 0.1831	R1 = 0.1383, wR2 = 0.3206
Absolute structure parameter		
Extinction coefficient	n/a	n/a
Largest diff. peak and hole	0.427 and -0.582 e.$Å^{-3}$	0.918 and -0.944 e.$Å^{-3}$
CCDC deposition number	2049236	2049240

a) b)

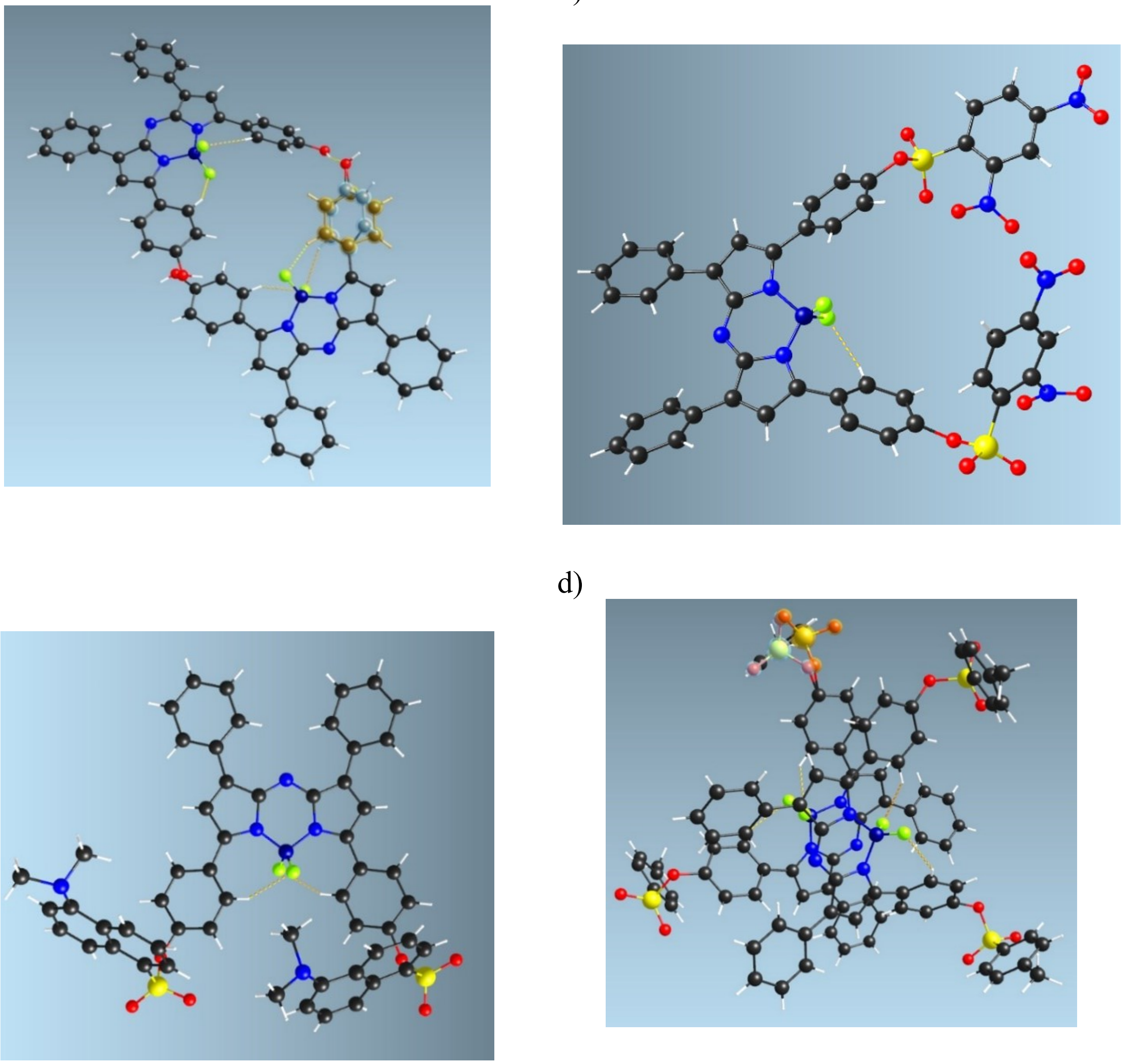

c) d)

Figure S17. X-ray crystal structures of (a) **BDP-OH**; (b) **BDP-1;** (c) **BDP-2** and (d) **BDP-3.**

2.3. Photophysical results

a)

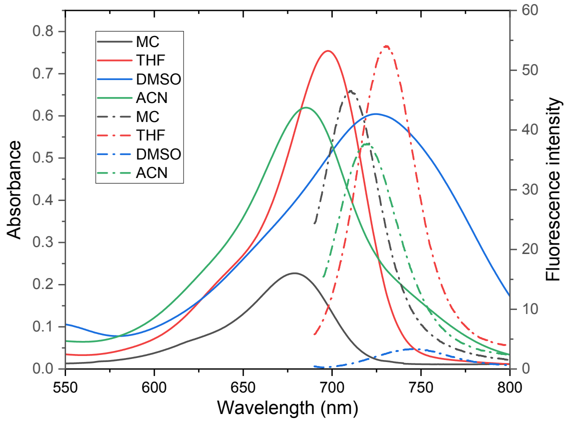

b)

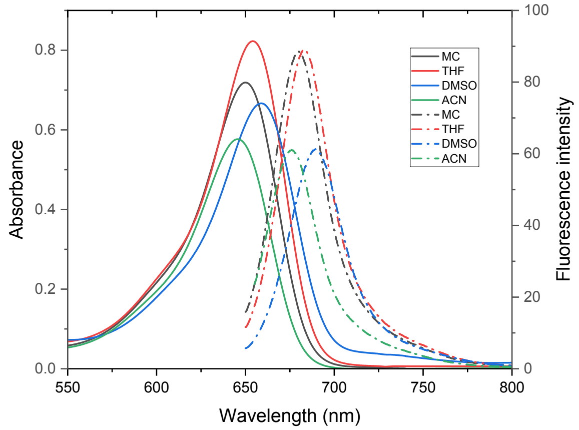

c)

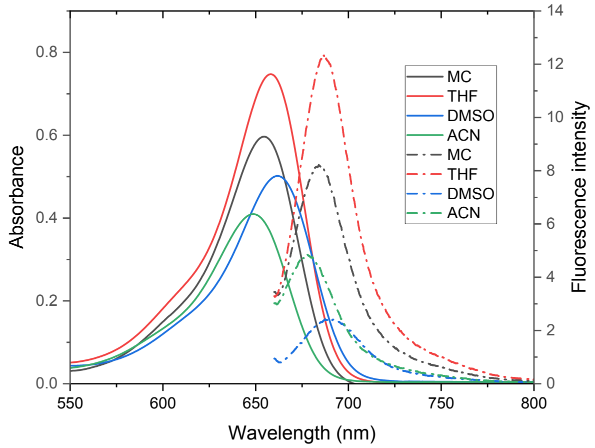

d)

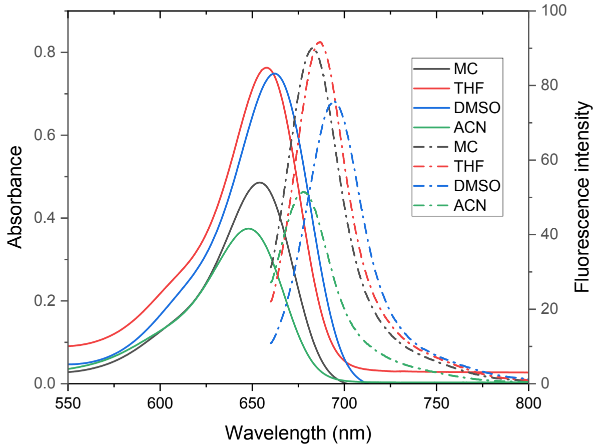

Figure S18. UV/Vis (solid line) and fluorescence emission (dash dot line) spectra of (a) **BDP-OH;** (b) **BDP-1;** (c) **BDP-2;** (d) **BDP-3** (10 μM) in various solvent (excitation at highest wavelength absorption).

a)

b)

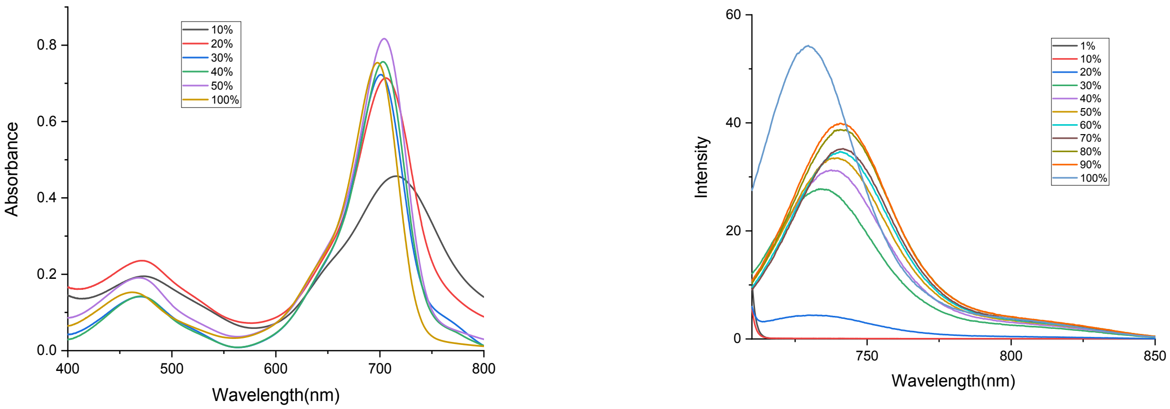

Figure S19. (a) UV/Vis and (b) fluorescence emission spectra of **BDP-OH** (10 µM) PBS 7.4 in the presence of THF (1-100%) (excitation at 700 nm).

Table S3. Photophysical properties of **BDP-OH, BDP-1, BDP-2, BDP-3**. [a]in MC, [b]in THF, [c]in DMSO, [d]in ACN.

	λ_{abs} (nm)	ε (L.mol^{-1}.cm^{-1})	λ_{ems} (nm)	Δ_ν (nm)	$\square_F$ (%)
**BDP-OH**	678.6[a]	22696	710.8	32.2	6.00
	697.8[b]	75408	731.0	33.2	2.58
	724.2[c]	60446	744.8	20.3	0.35
	685.4[d]	61956	719.4	34.0	1.72
**BDP-1**	650.0[a]	71898	680.0	30.0	3.65
	654.2[b]	82280	682.6	28.4	3.05
	658.8[c]	66661	689.4	30.6	3.09
	646.0[d]	57650	675.8	29.8	2.91
**BDP-2**	654.4[a]	59662	684.0	29.6	0.39
	658.2[b]	74746	686.2	28.0	0.46
	661.8[c]	50168	689.6	27.8	0.18
	648.1[d]	40943	678.0	29.9	0.30
**BDP-3**	653.0[a]	48503	682.6	29.6	5.10
	657.8[b]	76297	686.8	29.0	3.30
	662.0[c]	74887	693.6	31.6	3.46
	648.0[d]	37451	677.8	29.8	3.45

a) b)

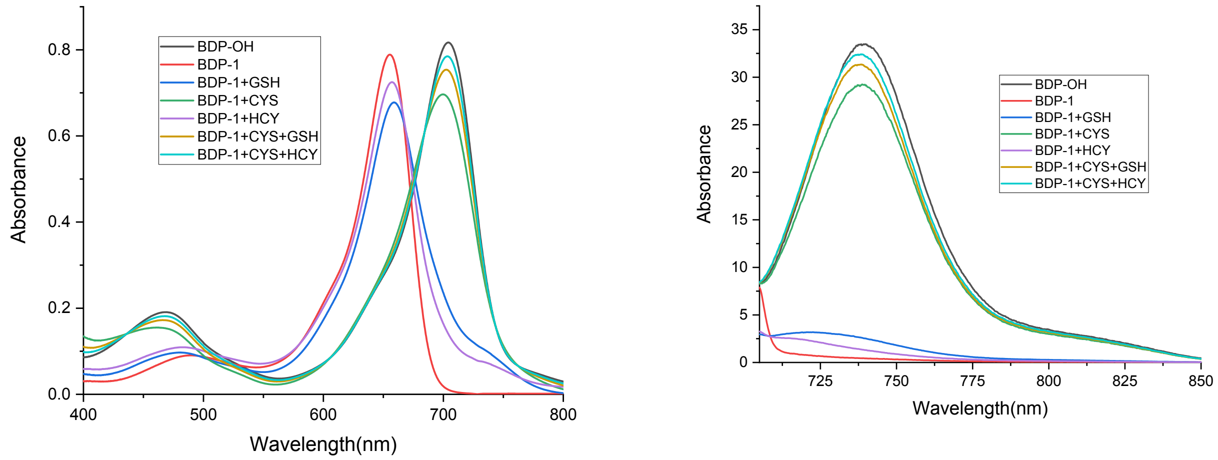

Figure S20. (a) UV/Vis and (b) fluorescence emission spectra of **BDP-1** (10 µM) in PBS (pH 7.4)/ THF (5/5) (excitation at 700 nm) in the presence of GSH, Hcy (1 mM), Cys (50 µM).

a)

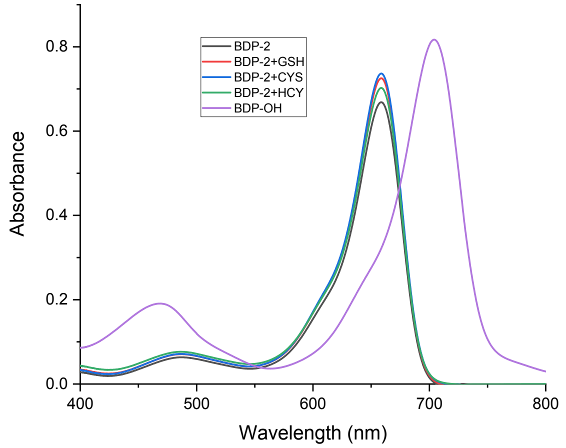

b)

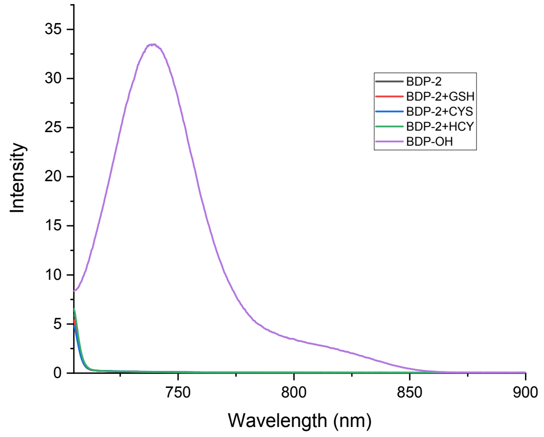

Figure S21. (a) UV/Vis and (b) fluorescence emission spectra of **BDP-OH** and **BDP-2** (10 μM) with GSH, Cys and Hcy (1 mM) in PBS (7.4 pH)/THF (5/5) (excitation at 700 nm).

a)

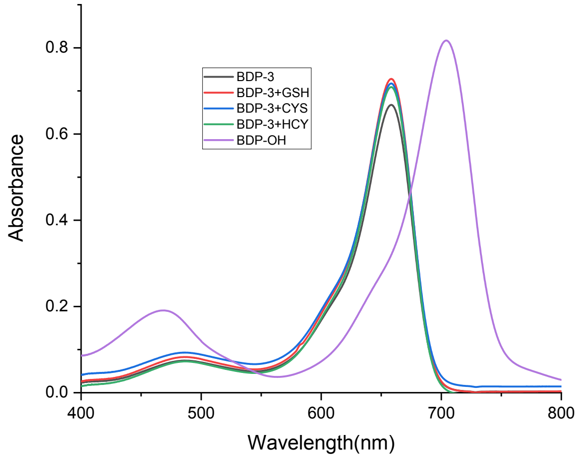

b)

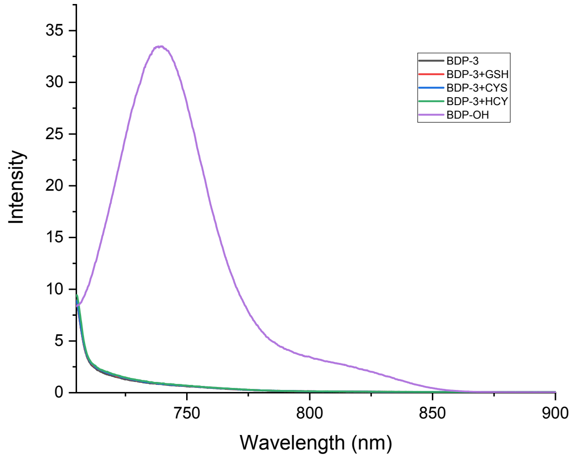

Figure S22. (a) UV/Vis and (b) fluorescence emission spectra of **BDP-OH** and **BDP-3** (10 μM) with GSH, Cys and Hcy (1 mM) in PBS (7.4 pH)/THF (5/5) (excitation at 700 nm).

a)

b)

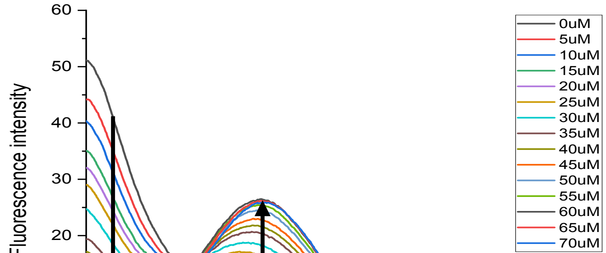

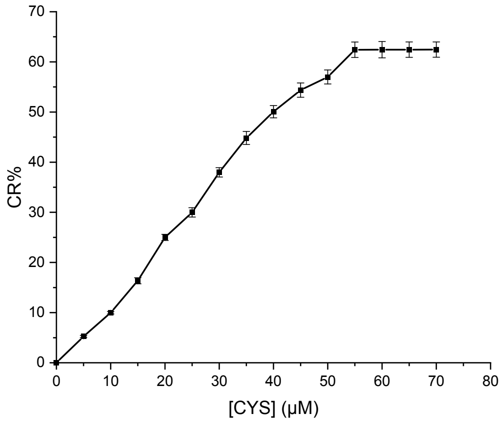

Figure S23. (a) Fluorescence emission spectra titrations of **BDP-1** (10 µM) in PBS (pH 7.4)/THF (5/5) (λ_{ex} = 675 nm) in the presence of Cys (0-70 µM) (slit 5/5 nm); (b) quantitative colorimetric response of **BDP-1** (10 µM) in the presence of Cys (0 - 80 µM) in PBS (pH 7.4)/THF (5/5).

2.4. DFT calculation results

Table S4. Excitation energy (eV) with significant oscillator strength (*f*) for the molecules predicted using different DFT functionals.

	**BDP-OH**	**BDP-1**	**BDP-2**	**BDP-3**	MAD*
Expt.	1.777 eV	1.892 eV	1.884 eV	1.885 eV	-
LSDA	**1.8095 eV**<br>f=0.7167	**1.9247 eV**<br>f=0.5406	**1.9274 eV**<br>f=0.6562	**1.9273 eV**<br>f=0.6926	**0.04**
BVP86	1.8244 eV<br>f=0.7278	1.9407 eV<br>f=0.5910	1.9398 eV<br>f=0.7022	1.9379 eV<br>f=0.7280	0.05
B3LYP	1.9902 eV<br>f=0.8616	2.0773 eV<br>f=0.5431	2.0824 eV<br>f=0.9066	2.0788 eV<br>f=0.9052	0.20
CAM-B3LYP	2.0669 eV<br>f=0.9341	2.1493 eV<br>f=0.9658	2.1337 eV<br>f=0.9627	2.1317 eV<br>f=0.9559	0.26
B3PW91	1.9911 eV<br>f=0.8636	2.0925 eV<br>f=0.7993	2.0871 eV<br>f=0.9100	2.0832 eV<br>f=0.9086	0.20
MPW1PW91	2.0203 eV<br>f=0.8885	2.1229 eV<br>f=0.9253	2.1035 eV<br>f=0.5232	2.1063 eV<br>f=0.9304	0.23
PBEPBE	1.8232 eV<br>f=0.7232	1.9443 eV<br>f=0.5893	1.9435 eV<br>f=0.7040	1.9409 eV<br>f=0.7275	0.05
HSEH1PBE	1.9991 eV<br>f=0.8711	2.1150 eV<br>f=0.8706	2.0962 eV<br>f=0.9220	2.0648 eV<br>f=0.9115	0.21
HCTH	1.8357 eV<br>f=0.7338	1.9552 eV<br>f=0.6196	1.9524 eV<br>f=0.7256	1.9500 eV<br>f=0.7489	0.06
TPSSTPSS	1.8670 eV<br>f=0.7602	1.9862 eV<br>f=0.6424	1.9833 eV<br>f=0.7491	1.9805 eV<br>f=0.7687	0.09
WB97XD	2.0712 eV<br>f=0.9402	2.1488 eV<br>f=0.9693	2.1335 eV<br>f=0.9667	2.1317 eV<br>f=0.9600	0.26
APFD	2.0092 eV<br>f=0.8785	2.1145 eV<br>f=0.9085	2.1029 eV<br>f=0.9191	2.0988 eV<br>f=0.9223	0.22
BhandHLYP	2.0996 eV<br>f=0.9710	2.1718 eV<br>f=1.0038	2.1570 eV<br>f=0.9990	2.1554 eV<br>f=0.9925	0.29
LC-wPBE	2.0345 eV<br>f=0.9488	2.1069 eV<br>f=0.9615	2.0922 eV<br>f=0.9607	2.0906 eV<br>f=0.9548	0.22
M06-2X	2.0766 eV<br>f=0.9255	2.1630 eV<br>f=0.9592	2.1472 eV<br>f=0.9564	2.1449 eV<br>f=0.9496	0.27

$$MAD = \left(\frac{1}{n}\right)\sum_{i=1}^{n}\left|E_{cal} - E_{expt}\right|$$

*Mean absolute deviations

Table S5. Calculated electronic transition energy, oscillator strength and orbital contribution obtained by DFT calculation (LSDA/gen). HOMO (H), LUMO (L).

	BDP-OH	BDP-1	BDP-2	BDP-3
S_0 - S_1	**1.8095 eV** f=0.7167<br>H → L 99%	1.2363 eV<br>f=0.0021	0.9460 eV f=0.0003	**1.9273 eV** f=0.6926<br>[H-1] → L 13%<br>H → L 87%
S_0 – S_2	2.1431 eV f=0.0214	1.2370 eV<br>f=0.0016	0.9462 eV f=0.0005	2.0971 eV f=0.1315
S_0 – S_3	2.1874 eV f=0.4861<br>[H-1] → L 95%	1.6555 eV f=0.0000	**1.9274 eV** f=0.6562<br>[H-3] → L 17%<br>[H-2] → L 83%	2.1597 eV f=0.3595<br>[H-2] → L 99%
S_0 – S_4	2.4405 eV f=0.0092	1.6555 eV f=0.0000	2.1023 eV f=0.1840	2.3844 eV f=0.0002
S_0 – S_5	2.5587 eV f=0.0009	1.7372 eV f=0.0001	2.1528 eV f=0.3581<br>[H-4] → L 99%	2.4006 eV f=0.0024
S_0 – S_6	2.5704 eV f=0.0011	1.7380 eV f=0.0004	2.3573 eV f=0.0000	2.5497 eV f=0.0003
S_0 – S_7		1.7820 eV f=0.0022		
S_0 – S_8		1.7826 eV f=0.0004		
S_0 – S_9		**1.9247 eV** f=0.5406<br>[H-1] → [L+2] 26%<br>H → [L+2] 73%		
S_0 – S_{10}		2.1271 eV f=0.3068		
S_0 – S_{11}		2.1379 eV f=0.3283<br>[H-2] → [L+2] 98%		
S_0 – S_{12}		2.1573 eV f=0.0000		

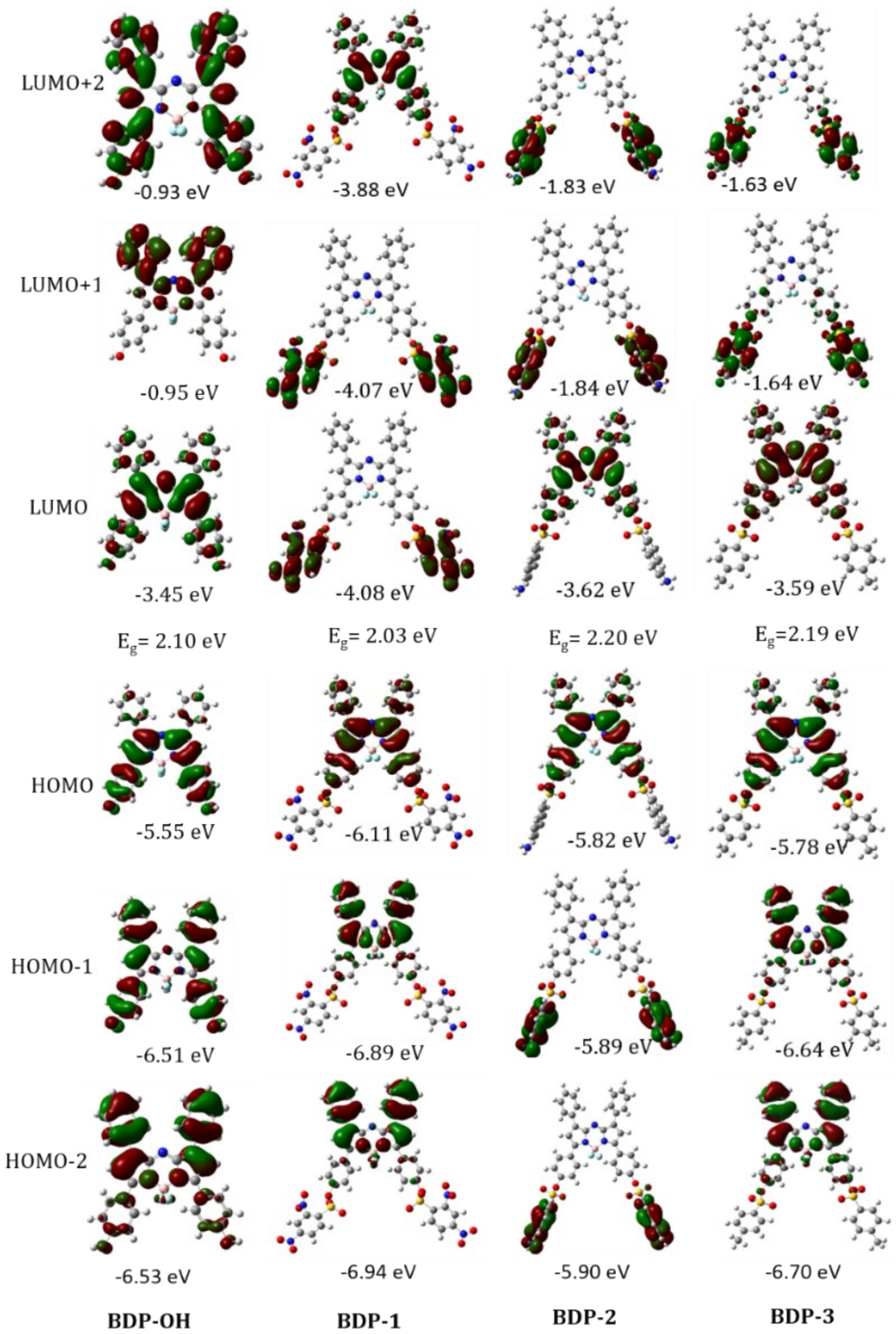

Figure S24. Molecular orbitals and energies (eV) of **BDP-OH**, **BDP-1**, **BDP-2**, and **BDP-3** in the ground state (S_0) obtained from the DFT calculations.

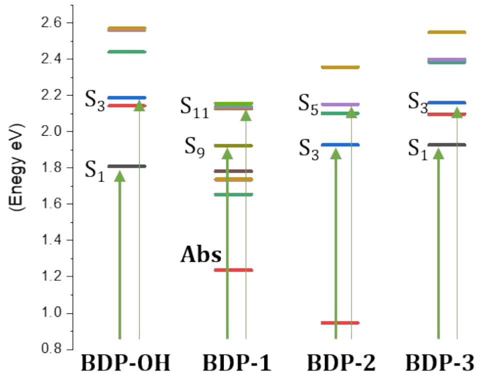

Figure S25. Electronic energy levels obtained by DFT calculation (LSDA/gen).

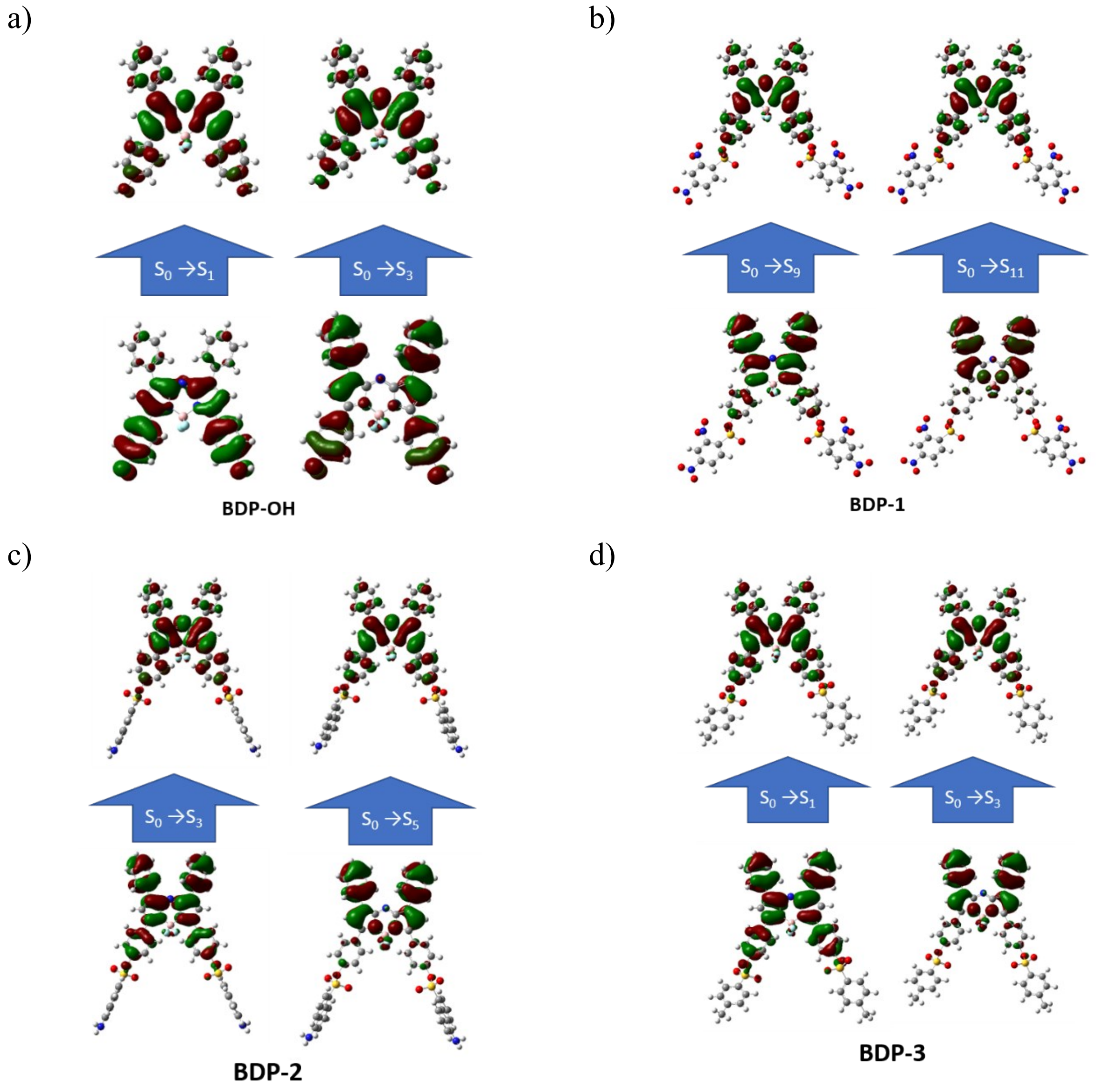

Figure S26. Natural transition orbitals (NTOs) of **BDP-OH** and **BDP-1** – **BDP-3** for given transitions.

Table S6: Ground state (S0) optimized geometries of all the molecules using B3LYP/gen. Gibbs energy (in Hartree) was calculated using B3LYP/gen.

**BDP-OH**	E=-1771.353918			**BDP-1**	E = -4148.22328		
C	-1.07176400	-1.14619400	0.03015000	C	-1.13933900	4.82722600	0.12986100
C	-1.07166600	1.14625300	-0.03021700	C	1.13920200	4.82727000	-0.12987200
C	0.65409000	-2.58499500	0.06833500	C	-2.56601600	3.10390100	0.29806200
C	-0.55218500	-3.34165200	0.02707800	C	-3.32666600	4.30543500	0.32348800
C	-1.63914800	-2.47239800	0.00582000	C	-2.46371900	5.39446600	0.22179900
H	-0.59564000	-4.41937800	0.02284800	H	-4.40099700	4.34591300	0.41023800
C	-1.63894900	2.47249900	-0.00584200	C	2.46356000	5.39456100	-0.22179900
C	0.65429900	2.58492400	-0.06837100	C	2.56594600	3.10400100	-0.29807500
C	-0.55192000	3.34166900	-0.02706400	C	3.32655000	4.30556400	-0.32349100
H	-0.59529700	4.41939800	-0.02278400	H	4.40088000	4.34608400	-0.41023500
N	0.32733000	1.25245300	-0.09150400	N	1.23841800	3.42857000	-0.20187800
N	0.32722300	-1.25250000	0.09140400	N	-1.23850200	3.42852100	0.20185700
B	1.23504000	-0.00006700	-0.00007900	B	-0.00002500	2.51838200	-0.00001400
F	2.07625000	0.13951000	1.16882200	F	0.24374300	1.68023600	1.15151000
F	2.07615500	-0.13973500	-1.16903700	F	-0.24376300	1.68023400	-1.15154200
C	-3.05967400	2.83751400	0.01935500	C	2.83067000	6.81359600	-0.21819200
C	-4.03367800	2.00828100	0.61932400	C	2.06012500	7.78179900	0.46427400
C	-3.47912700	4.06274200	-0.54753400	C	4.00093200	7.23673300	-0.88942000
C	-5.37710200	2.39648600	0.64990500	C	2.45177800	9.12418800	0.47310700
H	-3.73173000	1.06867800	1.06491900	H	1.16565700	7.47641000	0.99267300
C	-4.82212100	4.44652200	-0.51505000	C	4.38730400	8.57897900	-0.87908200
H	-2.75060600	4.70222000	-1.03627600	H	4.59326100	6.51260800	-1.44012800
C	-5.77848400	3.61457300	0.08488200	C	3.61427200	9.52958300	-0.19653400
H	-6.10966500	1.74875900	1.12203800	H	1.85175900	9.85244600	1.01015700
H	-5.12400700	5.38827900	-0.96339600	H	5.28510500	8.88497500	-1.40729200
H	-6.82225300	3.91238300	0.11095700	H	3.91548000	10.57252900	-0.18670500
C	-3.05990100	-2.83730800	-0.01934800	C	-2.83088300	6.81348700	0.21820000
C	-4.03385100	-2.00802100	-0.61933000	C	-2.06037500	7.78172300	-0.46425900
C	-3.47943700	-4.06249000	0.54757900	C	-4.00116100	7.23657500	0.88943300
C	-5.37730500	-2.39612700	-0.64988300	C	-2.45208000	9.12409700	-0.47308200
H	-3.73184000	-1.06845300	-1.06495600	H	-1.16589600	7.47637100	-0.99266000
C	-4.82246000	-4.44617200	0.51512200	C	-4.38758400	8.57880600	0.87910400
H	-2.75095700	-4.70200800	1.03632800	H	-4.59346200	6.51242400	1.44013600
C	-5.77876900	-3.61416900	-0.08482000	C	-3.61458800	9.52944400	0.19656100
H	-6.10982500	-1.74836000	-1.12202500	H	-1.85208800	9.85238200	-1.01012800
H	-5.12440900	-5.38789400	0.96349800	H	-5.28539600	8.88476500	1.40731600
H	-6.82256000	-3.91190200	-0.11087400	H	-3.91583500	10.57237900	0.18673900
C	1.99178800	-3.17948100	0.05963800	C	-3.15521900	1.75918700	0.34065800
C	3.11747000	-2.60253500	0.69410800	C	-2.52872500	0.65447100	0.95740000
C	2.16299400	-4.42488500	-0.59053400	C	-4.43708900	1.58034500	-0.23316900
C	4.35283500	-3.24474600	0.68068600	C	-3.16329100	-0.58959100	0.99665700
H	3.02118100	-1.65542400	1.20680700	H	-1.55314500	0.76556100	1.41023100
C	3.39998900	-5.06682000	-0.61474100	C	-5.07852000	0.34210900	-0.20012600
H	1.32758500	-4.87955300	-1.11178700	H	-4.92306200	2.40875300	-0.73604600
C	4.49624900	-4.47324500	0.02632800	C	-4.42389800	-0.72089200	0.41964000
H	5.21371900	-2.80737900	1.17259500	H	-2.69047700	-1.43996700	1.47337900
H	3.51093900	-6.01407700	-1.13607500	H	-6.05472400	0.19871000	-0.64577800
C	1.99203200	3.17933400	-0.05964300	C	3.15520100	1.75931000	-0.34067500
C	2.16330100	4.42469100	0.59060600	C	4.43706900	1.58051300	0.23317200
C	3.11768900	2.60238000	-0.69415100	C	2.52876000	0.65457600	-0.95743800
C	3.40032500	5.06656700	0.61485400	C	5.07854700	0.34230100	0.20012900
H	1.32791500	4.87936700	1.11189000	H	4.92300100	2.40893500	0.73606500
C	4.35308400	3.24453200	-0.68068800	C	3.16337600	-0.58946000	-0.99669700
H	3.02135700	1.65531200	-1.20691800	H	1.55318400	0.76563300	-1.41028300
C	4.49655600	4.47298200	-0.02625300	C	4.42397700	-0.72072000	-0.41965900
H	3.51131800	6.01378600	1.13624800	H	6.05474800	0.19893500	0.64579700
H	5.21394600	2.80715700	-1.17262900	H	2.69060200	-1.43985000	-1.47343600
N	-1.73112200	0.00005700	-0.00003200	N	-0.00008100	5.48580100	-0.00000400
O	5.76055800	5.05601900	-0.04909700	O	5.10633900	-1.98714900	-0.53512400
H	5.79656900	5.90414300	0.43334200	O	-5.10620800	-1.98734900	0.53510800

O	5.76022300	-5.05633900	0.04920500	C	6.37253000	-4.12641300	0.13246800
H	5.79619500	-5.90449400	-0.43318100	C	6.06274600	-5.28242400	-0.59675200
				C	7.70821600	-3.88651400	0.50082800
				C	7.06919300	-6.17528600	-0.97577600
				H	5.02936400	-5.48277400	-0.84966200
				C	8.72095500	-4.77938800	0.15915900
				C	8.38313200	-5.90829700	-0.58910500
				H	6.84086800	-7.06425300	-1.54839000
				H	9.74388500	-4.60386700	0.46456600
				C	-6.37240800	-4.12659100	-0.13250200
				C	-6.06261600	-5.28262500	0.59667700
				C	-7.70810200	-3.88665200	-0.50079900
				C	-7.06906600	-6.17547800	0.97571400
				H	-5.02922900	-5.48300100	0.84954200
				C	-8.72084800	-4.77950900	-0.15910800
				C	-8.38301600	-5.90845000	0.58910700
				H	-6.84073700	-7.06446500	1.54829500
				H	-9.74378700	-4.60395600	-0.46446400
				S	4.99507100	-3.10502400	0.66929700
				S	-4.99496600	-3.10518900	-0.66934700
				O	5.31339700	-2.50159000	1.96219200
				O	3.77100500	-3.88339800	0.48003800
				O	-5.31334000	-2.50171600	-1.96221200
				O	-3.77089000	-3.88356200	-0.48014900
				N	9.45618500	-6.84998000	-0.97361400
				N	-9.45607300	-6.85012100	0.97363500
				O	-10.60339300	-6.57807700	0.61664000
				O	-9.12905300	-7.84251600	1.62618000
				O	9.12917000	-7.84236000	-1.62618700
				O	10.60349300	-6.57797900	-0.61654800
				N	8.13532900	-2.68026400	1.24645700
				N	-8.13520800	-2.68036300	-1.24636700
				O	-7.89818300	-1.59028600	-0.72919600
				O	-8.74736000	-2.87053000	-2.29419500
				O	8.74740300	-2.87048900	2.29432000
				O	7.89839700	-1.59016000	0.72930100
	**BDP-2**	E = -3748.131097			**BDP-3**	E = -3409.519283	
C	-5.05229200	-1.12955000	0.19422500	C	4.19882800	-1.14414800	-0.06021900
C	-3.32827100	2.54826300	-0.43731000	C	4.19813400	1.14633700	0.06010600
C	-4.53284100	3.30355300	-0.51225800	C	2.47541300	-2.58052400	-0.13782400
C	-5.62026700	2.44657100	-0.36493800	C	3.67984500	-3.33924500	-0.11917800
H	-4.57529000	4.37080500	-0.66178700	C	4.76723200	-2.47084400	-0.07298700
C	-5.62116600	-2.44498000	0.36499000	H	3.72191800	-4.41671400	-0.14188900
C	-3.32919800	-2.54754000	0.43734300	C	4.76572700	2.47337700	0.07285300
C	-4.53406700	-3.30236600	0.51230300	C	2.47384400	2.58165700	0.13788500
H	-4.57696500	-4.36959700	0.66184300	C	3.67781000	3.34111100	0.11916300
N	-3.65380800	-1.22697900	0.26651400	H	3.71923200	4.41860400	0.14189600
N	-3.65337100	1.22782900	-0.26652200	N	2.79946800	1.25048200	0.12343300
B	-2.74390400	0.00027100	0.00001900	N	2.80021800	-1.24915200	-0.12342800
F	-1.90807600	-0.30236400	-1.13835500	B	1.88994900	0.00038300	0.00008500
F	-1.90801700	0.30263600	1.13842600	F	1.05300300	0.16964400	-1.16497900
C	-7.04156000	-2.80827500	0.38899600	F	1.05333500	-0.16939400	1.16531900
C	-8.01083200	-2.07739700	-0.33415600	C	6.18564900	2.83932600	0.05381900
C	-7.46532200	-3.93460300	1.13089500	C	7.15749000	2.02822200	-0.57396800
C	-9.35431700	-2.46529100	-0.31451500	C	6.60613800	4.04812900	0.65441400
H	-7.70528700	-1.21584100	-0.91461900	C	8.50046300	2.41787400	-0.59836300
C	-8.80859700	-4.31776900	1.14850300	H	6.85432000	1.10256900	-1.04708200
H	-6.74037800	-4.49428900	1.71377000	C	7.94879000	4.43297500	0.62832200
C	-9.76018100	-3.58478800	0.42451100	H	5.87901700	4.67320900	1.16339800
H	-10.08318100	-1.89586100	-0.88327200	C	8.90309700	3.61913300	0.00077000
H	-9.11444500	-5.18158700	1.73089400	H	9.23147700	1.78508100	-1.09250600
H	-10.80395600	-3.88327200	0.43664400	H	8.25207600	5.36156000	1.10228500
C	-7.04052700	2.81037900	-0.38894600	H	9.94651200	3.91839400	-0.02080400

C	-8.01004400	2.07992500	0.33430600	C	6.18738300	-2.83590700	-0.05407900
C	-7.46389500	3.93679000	-1.13094400	C	7.15875600	-2.02421400	0.57367100
C	-9.35338800	2.46830400	0.31465800	C	6.60859100	-4.04441100	-0.65477000
H	-7.70479200	1.21832300	0.91485600	C	8.50198100	-2.41300500	0.59792800
C	-8.80703200	4.32043800	-1.14856200	H	6.85503200	-1.09878100	1.04686000
H	-6.73875100	4.49615500	-1.71387900	C	7.95149300	-4.42839300	-0.62882100
C	-9.75886300	3.58787200	-0.42447600	H	5.88182100	-4.66993100	-1.16371600
H	-10.08244700	1.89920000	0.88349200	C	8.90533300	-3.61397000	-0.00131200
H	-9.11258200	5.18430900	-1.73103200	H	9.23263300	-1.77977200	1.09204300
H	-10.80253100	3.88673000	-0.43661500	H	8.25533500	-5.35675700	-1.10286100
C	-1.98647000	3.13956400	-0.50599400	H	9.94894300	-3.91256000	0.02014900
C	-0.86539400	2.47573800	-1.05089600	C	1.13400100	-3.17578900	-0.14510500
C	-1.82304000	4.46382000	-0.03096900	C	0.01990700	-2.58525200	-0.78105500
C	0.37457500	3.11512600	-1.11825200	C	0.96344300	-4.43199900	0.48620500
H	-0.96057200	1.46635600	-1.42641800	C	-1.21897500	-3.22943100	-0.78275000
C	-0.58928000	5.10984700	-0.09759700	H	0.12048200	-1.62960800	-1.27670200
H	-2.66149500	4.98195500	0.42066700	C	-0.27009900	-5.08212800	0.48566200
C	0.49453100	4.42119100	-0.64685400	H	1.79700000	-4.89089900	1.00617600
H	1.23865300	2.60911300	-1.53194300	C	-1.34885600	-4.46723500	-0.15499900
H	-0.46084700	6.11780800	0.27702200	H	-2.07654800	-2.78172900	-1.27061800
C	-1.98763600	-3.13934000	0.50604300	H	-0.39994000	-6.03781100	0.97830800
C	-1.82478600	-4.46381800	0.03140200	C	1.13206800	3.17610400	0.14527700
C	-0.86619800	-2.47580600	1.05055300	C	0.96073100	4.43227700	-0.48590200
C	-0.59127600	-5.11030100	0.09802000	C	0.01835100	2.58481700	0.78119100
H	-2.66350600	-4.98178600	-0.41993200	C	-0.27321500	5.08163100	-0.48525400
C	0.37353500	-3.11565900	1.11789300	H	1.79398500	4.89173300	-1.00586600
H	-0.96089200	-1.46630100	1.42586000	C	-1.22093100	3.22823500	0.78299900
C	0.49291500	-4.42191100	0.64687700	H	0.11953400	1.62919100	1.27674700
H	-0.46330600	-6.11844100	-0.27628200	C	-1.35157300	4.46602400	0.15537900
H	1.23786600	-2.60983000	1.53128000	H	-0.40371100	6.03725500	-0.97784200
N	-5.71059400	0.00080700	0.00002900	H	-2.07821000	2.77994700	1.27084500
O	1.74949000	-5.08796200	0.80749800	N	4.85707900	0.00129400	-0.00008900
O	1.75137900	5.08676600	-0.80752900	O	-2.61257500	5.13514500	0.25311900
S	2.73417900	-5.28922800	-0.52943500	O	-2.60952300	-5.13695400	-0.25277100
S	2.73619800	5.28768100	0.52931900	C	-4.96987600	6.03492900	-0.30363200
O	2.09598500	-6.26224900	-1.42613800	C	-5.99155000	5.21851700	0.19783200
O	3.09115200	-3.96473500	-1.04933100	C	-5.04116300	7.43179600	-0.19989300
O	2.09792600	6.26017900	1.42653700	C	-7.09206300	5.81516000	0.82044700
O	3.09356000	3.96305500	1.04861300	H	-5.92729700	4.14160000	0.09536200
C	4.13037200	6.03642300	-0.30437100	C	-6.14777300	8.00911900	0.42553400
C	5.25879900	6.42053000	0.50192100	C	-7.18834900	7.21338200	0.94537100
C	4.10494000	6.22321100	-1.67603400	H	-7.88657400	5.18682000	1.21142600
C	5.33881000	6.21060700	1.90794500	H	-6.20730800	9.09050000	0.50884100
C	6.36844900	7.04111900	-0.18173200	C	-4.96647300	-6.03774100	0.30373500
C	5.21962400	6.80187200	-2.32538700	C	-5.98838000	-5.22028800	-0.19555700
H	3.24087700	5.92066500	-2.24991800	C	-5.03793700	-7.43440100	0.19739100
C	6.46628500	6.60544700	2.60248400	C	-7.08932200	-5.81565400	-0.81862800
H	4.52347100	5.73972400	2.44037800	H	-5.92395700	-4.14357300	-0.09108800
C	7.50755600	7.47573300	0.58325600	C	-6.14498300	-8.01044300	-0.42845000
C	6.32220500	7.19830100	-1.59453800	C	-7.18581000	-7.21363400	-0.94613300
H	5.20211300	6.92091000	-3.40333800	H	-7.88401100	-5.18650300	-1.20793600
C	7.54388500	7.24344700	1.95130100	H	-6.20465400	-9.09165800	-0.51378300
H	6.52143500	6.43672100	3.67318200	S	-3.58141200	5.29161700	-1.10946300
H	7.18075300	7.60840200	-2.11481200	S	-3.57745000	-5.29607700	1.11011900
H	8.40702100	7.56382400	2.52818500	O	-2.94389400	6.26327800	-2.00663900
C	4.12862300	-6.03734000	0.30436500	O	-3.92748600	3.94710300	-1.58087600
C	5.25686300	-6.42188400	-0.50198800	O	-2.93942100	-6.26957200	2.00494700
C	4.10358700	-6.22322900	1.67615400	O	-3.92313300	-3.95247800	1.58441900
C	5.33651000	-6.21277800	-1.90816200	H	-4.24533600	-8.05105900	0.60444400
C	6.36671700	-7.04199200	0.18174400	H	-4.24877200	8.04762300	-0.60861000
C	5.21848400	-6.80142200	2.32556700	C	-8.38800000	7.85506200	1.60488200
H	3.23966500	-5.92038000	2.25009100	H	-8.08218000	8.59084900	2.35810700
C	6.46383700	-6.60794200	-2.60274800	H	-9.00713600	8.38285200	0.86752000
H	4.52099600	-5.74227000	-2.44065900	H	-9.01972000	7.10955600	2.09723000

C	7.50565100	-7.47702100	-0.58327800	C	-8.38596000	-7.85395200	-1.60606100
C	6.32087300	-7.19828800	1.59466400	H	-8.08071600	-8.58916800	-2.36006700
H	5.20128700	-6.91973800	3.40360300	H	-9.00526500	-8.38210600	-0.86909600
C	7.54163500	-7.24551200	-1.95145600	H	-9.01735000	-7.10758500	-2.09752800
H	6.51873600	-6.43981700	-3.67355300				
H	7.17957800	-7.60805400	2.11494100				
H	8.40464800	-7.56615800	-2.52837400				
N	8.60556100	8.06897100	-0.07647000				
H	8.34122900	8.76255500	-0.76688700				
H	9.25615100	8.49154600	0.57539500				
N	8.60383800	-8.06986400	0.07655500				
H	8.33964400	-8.76311100	0.76736400				
H	9.25427800	-8.49279800	-0.57523200				
